# Clinical Use of Molecular Biomarkers in Canine and Feline Oncology: Current and Future

**DOI:** 10.3390/vetsci11050199

**Published:** 2024-05-02

**Authors:** Heike Aupperle-Lellbach, Alexandra Kehl, Simone de Brot, Louise van der Weyden

**Affiliations:** 1 Laboklin GmbH&Co.KG, Steubenstr. 4, 97688 Bad Kissingen, Germany; aupperle@laboklin.com (H.A.-L.); kehl@laboklin.com (A.K.); 2 School of Medicine, Institute of Pathology, Technical University of Munich, Trogerstr. 18, 80333 München, Germany; 3 Institute of Animal Pathology, COMPATH, University of Bern, 3012 Bern, Switzerland; simone.debrot@unibe.ch; 4 Wellcome Sanger Institute, Wellcome Genome Campus, Hinxton, Cambridge CB10 1SA, UK

**Keywords:** genetic, mutation, tumour, diagnostic, prognostic, predictive, somatic, germline, dog, cat

## Abstract

**Simple Summary:**

Molecular biomarkers in cancer are measurable genomic alterations that can indicate the risk of developing neoplasia, the presence of neoplastic cells, patient outcome, and/or a likely response to therapy. This review discusses the different uses of molecular biomarkers in the veterinary clinic and how their presence can be determined. In particular, we showcase which genomic alterations are currently used to date as molecular biomarkers in the clinic and for what purposes. We also look at biomarkers that are currently being developed and show promise for clinical use. Finally, we consider the important factors that allow a molecular biomarker to move from the research laboratory to the clinic. This review aims to enable veterinarians to understand the benefits of molecular biomarkers in delivering precision veterinary care to dogs and cats with cancer.

**Abstract:**

Molecular biomarkers are central to personalised medicine for human cancer patients. It is gaining traction as part of standard veterinary clinical practice for dogs and cats with cancer. Molecular biomarkers can be somatic or germline genomic alterations and can be ascertained from tissues or body fluids using various techniques. This review discusses how these genomic alterations can be determined and the findings used in clinical settings as diagnostic, prognostic, predictive, and screening biomarkers. We showcase the somatic and germline genomic alterations currently available to date for testing dogs and cats in a clinical setting, discussing their utility in each biomarker class. We also look at some emerging molecular biomarkers that are promising for clinical use. Finally, we discuss the hurdles that need to be overcome in going ‘bench to bedside’, i.e., the translation from discovery of genomic alterations to adoption by veterinary clinicians. As we understand more of the genomics underlying canine and feline tumours, molecular biomarkers will undoubtedly become a mainstay in delivering precision veterinary care to dogs and cats with cancer.

## 1. Introduction

It is well established that the diagnosis of tumours in dogs and cats may require inspection, palpation, as well as diagnostic imaging (such as X-rays, ultrasound, MRI, and CT scans), followed by sampling of the mass for cytological or histological analysis (with further immunohistochemical characterisation if relevant). In human medicine, molecular characterisation is also a vital part of the diagnostic workup for many neoplasms. Furthermore, molecular diagnostics may provide a wealth of information to aid clinical decision-making, such as prognosis and therapeutic options. For example, a recent study from the Cancer Programme of the ‘100,000 Genomes Project’ analysed the genomic and clinical data of 13,880 tumours and demonstrated that linking molecular characterisation of tumours to ‘real-world’ clinical data such as survival analysis, allows the identification of cancer genes that affect prognosis, as well as furthering our understanding of how cancer genomics can impact patient outcomes [1].

The clinical use of molecular biomarkers in human medicine is only possible because of the development of technologies that allow reliable identification and interpretation of these markers, the presence of a high-quality reference genome, and robust investigations into the applicability of these markers in guiding diagnostic, therapeutic, and/or prognostic clinical decisions. Over the last ten years, molecular-based studies of canine and feline tumours have dramatically increased. Characterisation of both the genetic and epigenetic alterations of tumours has been performed, from single tumour type to pan-cancer studies, using various technologies. This review looks at which molecular alterations in dogs and cats are used in the clinical setting as oncological molecular biomarkers for diagnostic, therapeutic, predictive, or screening purposes to date. We also examine what is needed to take a molecular biomarker from ‘bench to clinic’ and the important factors that must be considered.

## 2. Molecular Alterations in Cancer

Cancers develop due to the accumulation of genetic and epigenetic alterations, induced by aging, radiation, ultraviolet light, mutagenic chemicals/toxins, oxygen radicals, viruses, and other factors (reviewed in [2]). Genetic alterations are aberrations that directly affect the DNA, resulting in a change (‘mutation’) in the nucleotide sequence. These changes, which constitute the ‘cancer genome’, can involve small or large regions of DNA.

### 2.1. Types of Molecular Alterations

**Figure 1:**
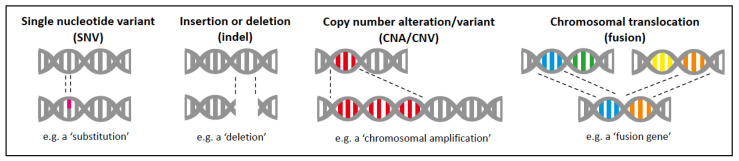
Types of molecular alterations. Single nucleotide variants (SNVs) occur when a single nucleotide is altered. They could be a substitution of one nucleotide for another (as shown in the example), a loss (deletion) of a nucleotide, or a gain (insertion) of a nucleotide. Insertion or deletion of up to 50 adjacent nucleotides are collectively termed indels. Structural variants are the result of genomic rearrangements and can include the gain of all or part of a chromosome (known as chromosomal amplification, as shown in the example) or the loss of all or part of a chromosome (known as chromosomal deletion). Genomic rearrangements also include chromosomal translocations, where a portion of a chromosome moves to another chromosomal location (which can result in a fusion gene, as shown in the example).

Molecular alterations can occur at the level of a single nucleotide (‘single nucleotide variants’, SNVs), such as substitutions, deletions, or insertions (Figure 1). This can result in a change in amino acid (such as caused by a substitution) or alter the entire reading frame and result in a truncated or nonsense protein (such as caused by a deletion or insertion). If the alteration involves adjacent nucleotides, they are referred to as ‘multi-nucleotide variants’ (MNVs). Small insertions or deletions (up to 50 nucleotides) are termed ‘indels’ (Figure 1). Alterations larger than 50 nucleotides are classified as ‘structural variants’. These can include genomic rearrangements, such as chromosomal deletions (loss of part or all of the chromosome) and chromosomal amplifications (gain of part or all of a chromosome; Figure 1), and are collectively known as either ‘copy number alterations’ (CNAs; when they occur in the tumour) or ‘copy number variants’ (CNVs; when they occur in the normal tissue). In such cases, it is interesting to identify the loss of tumour suppressor genes (TSGs) located within deletion regions and the gain of oncogenes located within amplification regions. Genomic rearrangements also include chromosomal translocations, where a portion of a chromosome moves to another chromosomal location (which can produce a ‘fusion gene’ if the new location brings together two genes; Figure 1). Other molecular alterations that can occur in cancer are epigenetic aberrations, which ‘modify’ rather than alter the DNA sequence. These aberrations, which constitute the ‘cancer epigenome’, include changes in DNA methylation, histone modification, chromatin remodelling, and non-coding RNA regulation (reviewed in [3]), and can regulate gene activity/expression.

### 2.2. Somatic versus Germline Alterations

Molecular alterations must be considered at two levels: somatic and germline. Somatic alterations are those found only in the tumour cells (i.e., the soma), and germline alterations are those present in all the cells of the body (i.e., present in the germ cells and thus inherited). Somatic alterations consist of ‘driver genes/mutations’, which confer a growth advantage to the tumour cells and thus have been positively selected during the development of the tumour, and ‘passenger genes/mutations’, which neither confer a growth advantage nor contribute to tumour development [4]. In humans, most cancers have more than one driver gene/mutation [5], and while some are frequently mutated across numerous tumour types, others occur more rarely and/or are only found in a particular tumour type [6]. Driver genes/mutations represent diagnostic, therapeutic, and/or prognostic biomarkers.

Germline alterations, present in every cell in the body, are important in cancer if they play a role in the predisposition to tumourigenesis. For example, Li-Fraumeni syndrome is a rare hereditary cancer predisposition syndrome in humans caused by a germline mutation in the *TP53* TSG, and affected individuals have a greatly increased risk of developing a wide range of cancers [7]. Thus, identification of germline predisposition genes/mutations is of clinical relevance, as it allows for increased clinical monitoring of the individual and/or the use of predictive measures where possible (such as mastectomy and oophorectomy in females with germline mutations in the TSGs, *BRCA1/2*).

Importantly, an increasing number of genes have been identified that can undergo either a somatic mutation or be associated with germline cancer susceptibility syndromes. Some well-established examples include *TP53*, *BRCA1*, and *BRCA2*.

### 2.3. Tissues/Fluids for Molecular Analysis

Molecular analyses can be performed on various body tissues and fluids (reviewed in [8]). The most common source for sampling tumour cells includes fine needle aspirates or tissue samples (such as through biopsies or surgical excisions). Tissue samples are typically placed in fixatives (such as formalin) to preserve the tissue architecture and allow histopathological analyses on the formalin-fixed paraffin-embedded samples (FFPE). Nucleic acids (DNA and RNA) can be isolated from FFPE samples for molecular analyses. However, it is important to note that formalin can have a range of detrimental effects on nucleic acids, especially if the tissues are stored there too long (12–24 h is generally considered optimal to minimise adverse effects on the DNA) [9]. Thus, although fresh or ‘fresh frozen’ tissue (where the issue is immediately placed into liquid nitrogen) is optimal, the difficulty in obtaining such samples means that the vast majority of analyses are performed on FFPE samples, and with great success [10].

Other sources of tumour cells for molecular analyses are bodily fluids, grouped under the term ‘liquid biopsy’. Examples include blood, saliva, urine, and procedure-induced fluids, such as bronchial washes and peritoneal effusions. Liquid biopsies can capture circulating tumour cells, circulating cell-free DNA (cfDNA), circulating tumour DNA (ctDNA), nucleosomes, and microRNAs, which can serve as a source of diagnostic, therapeutic, and/or prognostic biomarkers (reviewed in [8]). A recent study comparing liquid versus tissue biopsy for detecting actionable alterations in patients with advanced cancer found there were some limitations in assays using ctDNA, specifically a higher rate of false negatives due to ‘non-shedding’ of the tumour, low levels of plasma DNA analysed, or a low tumour fraction present in the sample [11]. However, it was concluded that there was still sufficient evidence for the clinical utility of liquid biopsies for capturing actionable alterations in patients with advanced cancer, either when tissue is not available or as a complementary approach to tissue-based strategies [11].

For molecular analysis of germline alterations, either for assessment of germline predisposition alleles or to ‘remove’ them from the analysis to allow assessment of somatic mutations in the tumour, healthy/non-tumour tissue can be used if available, or more typically, peripheral blood is used, and nucleic acids extracted from the white blood cells.

### 2.4. Tools for Molecular Analysis

There is an extensive range of techniques (‘tools’) available for molecular analyses of tissue/body fluid samples (reviewed in [8,12,13]), and the choice of which to use is dependent upon several factors. For example:

- Sample type (tissue versus body fluid, formalin-fixed versus fresh)
- Nucleic acids being analysed (DNA versus RNA)
- Type of genomic aberration being detected (SNVs, MNVs, CNAs, methylation)
- Cost (use of expensive equipment and highly specialised personnel/staff)
- Availability of technical expertise (computational analyses and interpretations)

The most common techniques currently used in clinical laboratories are DNA-based polymerase chain reaction (PCR)-based methods due to their sensitivity in detection levels, low cost relative to other methods, and comparative ease of use/analysis of the results. However, it should be noted that prior knowledge of the relevant DNA sequence/mutation/variant being assessed is a pre-requisite for the use of PCR. In contrast, techniques that are gaining traction include next-generation sequencing-based methods due to their ability to detect a large number and type of molecular aberrations from a single sample. For example, whole genome sequencing can determine all the nucleotides of the genome of an organism at a single time. Artificial intelligence (AI)-based histology is also rapidly evolving due to its power to identify and objectively measure tissue biomarkers (reviewed in [8]).

## 3. Classes of Biomarkers

The FDA-NIH Biomarker Working Group defines a biomarker (‘biological marker’) as “a defined characteristic that is measured as an indicator of normal biological processes, pathogenic processes, or biological responses to an exposure or intervention, including therapeutic interventions. Biomarkers may include molecular, histologic, radiographic, or physiologic characteristics” [14]. The present review focuses on molecular biomarkers in cancer.

### 3.1. Diagnostic Molecular Biomarkers

Diagnostic biomarkers are those used to identify or confirm the presence of a tumour in an animal and can also be used to determine the type of tumour. The usefulness of diagnostic biomarkers lies in the fact that a correct diagnosis allows for informed treatment and, thus, the best possible chance of survival. Diagnostic markers are typically used for symptomatic patients; therefore, when used in asymptomatic patients, they are more accurately termed ‘screening’ biomarkers (see Section 3.4 below).

### 3.2. Prognostic Molecular Biomarkers

Prognostic biomarkers are those used to predict a disease’s course and thus provide information about the patient’s overall cancer outcome [15]. As they are used to estimate the patient’s health outcome, typically in terms of length of survival, prognostic biomarkers can be used to guide aspects of patient care. They can also be considered covariates for patient stratification, such as when deciding whether to enroll a patient in a clinical trial. When establishing a molecular alteration as a prognostic marker, it must be objectively measured and evaluated among patients with the same tumour type. In addition, other parameters must often be considered, together with the molecular alteration, to provide valid prognostic information. Human medicine is increasingly using AI to optimally collate all these parameters [16,17,18].

### 3.3. Predictive Molecular Biomarkers

Predictive biomarkers (also termed ‘therapeutic markers’) are increasingly pivotal in optimising cancer treatment and the delivery of personalised medicine in humans. Hopefully, this is a trend that will soon follow in veterinary medicine. They are based on knowledge of specific tumour alterations or germline genetic variants that confer a pattern of sensitivity to specific therapeutic agents. Predictive markers aim to give information about the effect of a specific therapeutic intervention [15], with cancer patients classified as probable responders or non-responders. Thus, predictive biomarkers are employed to guide the use of specific treatments/therapies. In the case of targeted cancer treatments, where the antibody or small molecule inhibitor targets a specific genomic alteration, it is essential to perform a predictive biomarker assay to identify whether the individual’s tumour cells have that specific genomic alteration and thus will potentially benefit from the therapy [19]. Although predictive biomarkers are of most interest to veterinarians and pet owners, screening dog and cat tumours for the presence of these biomarkers is not yet a routine procedure. This is due to the current limited availability of targeted therapies in veterinary medicine, and the difficulties in accessing and financing human-targeted drugs for off-label use can make it prohibitive for many individuals.

### 3.4. Screening Molecular Biomarkers

Screening biomarkers can be used in two settings. Firstly, they can be used in asymptomatic individuals to ‘screen’ for the presence of a tumour as part of general health surveillance. This can aid in the early detection of a tumour before the onset of clinical manifestations, offering a better chance of successful treatment. This is particularly relevant for dog and cat breeds with a known increased risk of disease. Secondly, they can be used in cancer patients to ‘monitor’ the overall tumour burden to detect potential worsening of the disease, such as an increase in tumour size due to recurrence or resistance to treatment and/or the development of metastases (providing the molecular alterations of the tumour do not evolve over time). In the case of germline alterations, screening molecular biomarkers can be used to determine breeding strategies (so as to avoid breeding individuals carrying a tumour predisposition genomic alteration). However, one must always keep in mind that breeding against one genetic marker can lead to the accumulation of other undesirable genetic markers and the associated phenotypes. As such, the desired effect of a decrease in tumour occurrence may be overshadowed by the release of new diseases. Particularly in small breeding populations, inaccurate selection during breeding management can cause further bottlenecks in the breeding population, with detrimental consequences.

## 4. Somatic Molecular Alterations Currently Tested in the Clinical Setting

There are currently four ‘assessments’ of specific somatic molecular alterations that can be tested in the clinical setting of veterinary medicine. These are alterations in the *BRAF* gene, alterations in the *KIT* gene, rearrangements of the antigen receptor genes, and alterations in panels of known cancer genes. These are discussed below, regarding their context in human cancer and how they are utilised in veterinary medicine, specifically in dogs and cats.

### 4.1. BRAF

**Figure 2:**
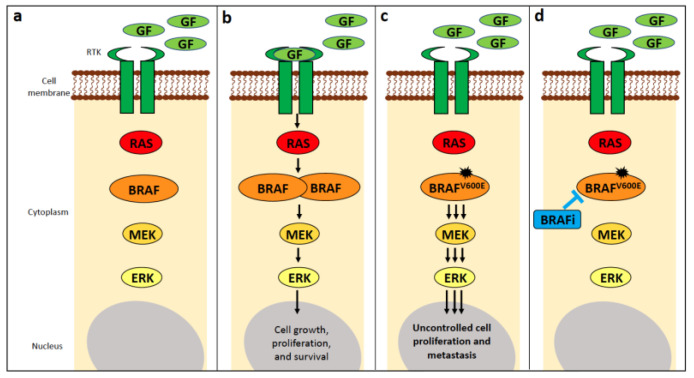
BRAF signalling. (**a**) BRAF functions in the mitogen-activated protein kinase (MAPK) signalling pathway, downstream of RAS. (**b**) Extracellular proliferative signals, such as growth factors (GF), activate receptor tyrosine kinases (RTKs), which in turn activate the RAS family. This induces dimerisation and activation of BRAF, leading to a signalling cascade of kinases, from MEK to ERK. Activated ERK can stimulate transcription, resulting in cell growth, proliferation, and survival. (**c**) Activating mutations in *BRAF*, such as *BRAF*^V600E^ confer constitutive activation of the MAPK pathway and result in uncontrolled cell proliferation and, in some cases, metastasis. (**d**) Small-molecule BRAF inhibitors (BRAFi) selectively target BRAF and thus block mutant BRAF-induced activation of the MAPK signalling pathway that regulates the proliferation and survival of the tumour cells.

BRAF belongs to the “rapidly accelerated fibrosarcoma” (RAF) family of mammalian cytosolic serine/threonine kinases, comprising ARAF, BRAF, and CRAF, which function in the mitogen-activated protein kinase (MAPK) signalling pathway downstream of RAS (reviewed in [20]) (Figure 2a). Extracellular proliferative signals (ligands) activate receptor tyrosine kinases (RTKs), which in turn activate GTPases belonging to the RAS family (i.e., KRAS, NRAS, and HRAS), which then induce dimerisation (and activation) of the RAF family members (Figure 2b). RAF activation leads to a signalling cascade of kinases, from MEK1/2 (mitogen-activated protein kinase kinase) to ERK1/2 (extracellular signal-regulated kinase), and phosphorylated ERK can stimulate transcription (Figure 2b). Activating mutations in components of this signalling cascade confer constitutive activation of the MAPK pathway and promote oncogenic transformation (Figure 2c). Although all three RAF kinases play essential roles in normal mammalian cells, it is predominantly BRAF that is altered in cancer.

#### 4.1.1. BRAF Alterations in Human Cancer

Analysis of over 80,000 human tumours found somatic *BRAF* mutations present in 4.6% of samples, most commonly occurring in hairy cell leukaemia (72%), thyroid cancer (45%), melanoma (36%), histiocytosis (19%), colorectal cancer (12%), and non-hairy cell leukaemia mature B cell neoplasms (3%) [21,22]. The *BRAF* p.V600E mutation is by far the most common RAF mutation, accounting for 46% of all *BRAF* alterations in the study of over 80,000 patients [21,22]. The *BRAF* p.V600E mutation results in the mutant BRAF protein mimicking the conformational changes that occur after dimerisation and thus becoming constitutively active despite still being a monomer and not having been activated by RAS (Figure 2c). Indeed, the mutant BRAF protein has a 500–700-fold increase in kinase activity relative to the wild-type BRAF protein [23].

The small molecule BRAF inhibitors (BRAFi), vemurafenib, dabrafenib, and encorafenib, are used in the treatment of human patients with *BRAF*-mutant melanoma, non-small cell lung cancer, anaplastic thyroid cancer, glioma, and colorectal cancer [24,25,26,27,28], as they selectively target BRAF and thus interfere with the MAPK signalling pathway that regulates the proliferation and survival of the tumour cells (Figure 2d).

#### 4.1.2. BRAF Alterations in Canine Cancer

Studies using a range of molecular technologies, from whole-exome sequencing to PCR of exon 15 of *BRAF* to droplet digital PCR of specific nucleotides in *BRAF*, have reported the *BRAF* p.V595E mutation in 40–87% of canine urinary bladder urothelial carcinoma (UC) and 60–85% of canine prostate carcinoma (PC) [29,30,31,32,33,34,35,36]. Importantly, this mutation has not been found in non-neoplastic (healthy) bladder and prostate tissue, as well as in dogs with urinary bladder cystitis, prostatitis, prostatic hyperplasia, prostatic metaplasia, or post-castration prostatic atrophy [29,31,37]. The mutation has also not been observed in germline samples of canines with UC (*n* = 96) [31].

In contrast to humans, the *BRAF* p.V595E mutation rarely occurs in other canine tumour types. A study of 667 canine tumour specimens found that apart from UC and PC, the *BRAF* p.V595E mutation was only present in a few tumour types and at low frequency, specifically pulmonary carcinoma (*n* = 1/18), oral squamous cell carcinoma (*n* = 2/18), melanoma (*n* = 3/54), melanocytoma (*n* = 3/18), glioma (*n* = 2/13) and peripheral nerve sheath tumour (*n* = 2/9), but no haematopoietic tumours (*n* = 245) or sarcomas (*n* = 160) [30].

**Figure 3:**
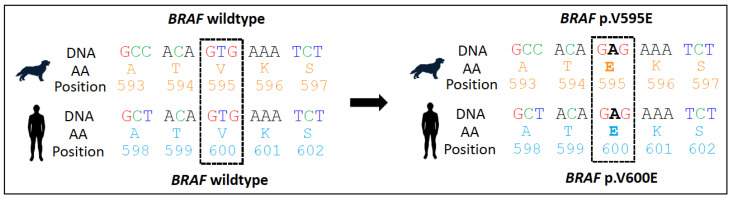
The canine *BRAF* p.V595E mutation. The *BRAF* p.V595E mutation in canine tumours involves a T > A nucleotide substitution, and results in an amino acid substitution of glutamic acid (V) to valine (E) at amino acid (AA) position 595 of the BRAF protein. This is the equivalent of the T > A nucleotide substitution in human BRAF, resulting in the *BRAF* p.V600E mutation in human tumours.

The *BRAF* p.V595E missense mutation involves a nucleotide substitution of T to A at position 1349 of the *BRAF* gene and results in an amino acid substitution of glutamic acid (V) to valine (E) at position 595 of the BRAF protein (also known as *BRAF* p.V588E if using the canonical transcript ENSCAFT00000006305.5). This is the equivalent of the human *BRAF* p.V600E hotspot mutation in human cancers (Figure 3).

#### 4.1.3. Use as a Diagnostic Biomarker in Canines

**Figure 4:**
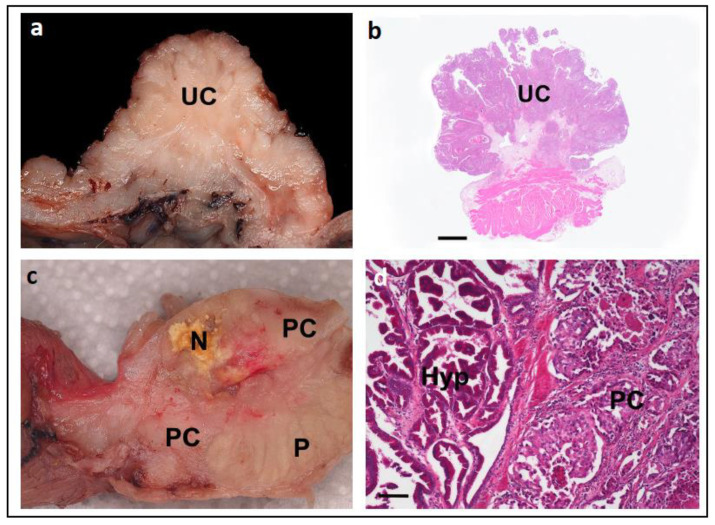
Canine urothelial carcinoma and prostate carcinoma. (**a**) Gross picture of a papilliform growing urothelial carcinoma (UC; 3.5 × 1.5 × 1.5 cm in size) in the urinary bladder of a 12-year-old male mixed breed dog (cross-section of a formalin-fixed sample). (**b**) Histology of a papilliform growing UC in the urinary bladder of a 6-year-old mixed-breed dog (HE stain, bar = 2.5 mm). (**c**) Gross picture of a longitudinal cut through the prostate of a Pinscher of unknown age with areas of prostate carcinoma (PC) with necrosis (N) besides normal glandular prostate tissue (P). (**d**) Histologically, the PC from an 11-year-old mixed breed dog is close to the hyperplastic glandular epithelium (Hyp) (HE stain, bar = 50 µm).

Canine UC and PC are characterised by local invasion and high rates of metastases. Clinical symptoms of these genitourinary neoplasms can include haematuria, stranguria, and/or incontinence. However, these symptoms are not specific and are also found in non-neoplastic conditions such as cystitis and prostatitis [38,39,40]. The gold standard for diagnosis of UC or PC, to definitively distinguish between these neoplastic and non-neoplastic conditions, is through cytology or histopathological evaluation of the tissue by way of a biopsy (Figure 4a).

However, obtaining a biopsy is not always possible. For example, elderly dogs have an increased risk for anaesthesia, there may be limited access to ultrasound equipment, and/or owners/clinicians may be discouraged from this procedure due to its invasive nature, the potential risk of seeding tumour cells, and the associated cost. In addition, the anatomical location of these tumours can make it challenging to obtain sufficient amounts of tissue for diagnosis, and sometimes the cytological and histological findings can be inconclusive (such as due to pleomorphism in severe inflammation and/or squeeze artefacts).

**Figure 5:**
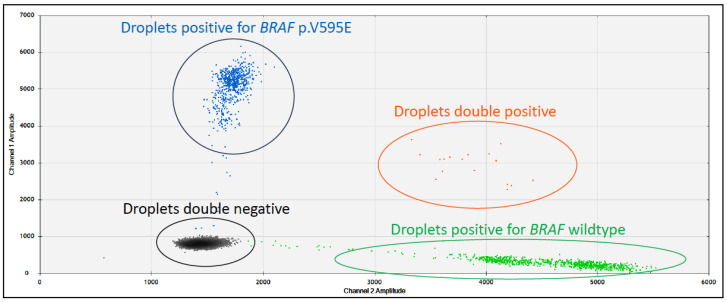
ddPCR result for *BRAF* p.V595E in urine from a 10-year-old castrated female Jack Russell Terrier with urinary bladder UC. Results are shown as 2D plots.

Thus, the desire for a non-invasive means of diagnosing these cancers led to the finding that the *BRAF* p.V595E mutation could be detected from neoplastic cells or cell-free DNA (cfDNA) in the urine of dogs with UC. It is now a diagnostic tool [29,37]. Typically performed by droplet digital PCR, detection of the *BRAF* p.V595E mutation in the urine of dogs with UC has been shown to have a sensitivity of 85% (*n* = 22/26) and a specificity of 100% (*n* = 37/37) (Figure 5) [29]. However, for routine diagnostics, it is important to note that in the case of canine UC, the incidence of the *BRAF* p.V595E mutation depends on the breed [41,42]. For example, as the incidence of this mutation in UC is so high in high- and low-legged terriers, it is recommended as a screening test from the age of 7 years in these breeds [31], as they have an exceptionally high prevalence of developing this tumour [43]. However, although the incidence is significantly lower in other dog breeds (~40–50% cases of UC), it can still be helpful as a first, non-invasive diagnostic step [37]. Interestingly, a recent case report identified the *BRAF* p.V595E mutation by digital PCR in a urine sample from a dog with follicular cystitis and a flat urothelial lesion with atypia [44], suggesting there is a continuum from dysplasia to carcinoma *in situ* to invasive carcinoma, and the mutation of *BRAF* may be an early driver event.

In non-*BRAF*-mutated UC cases, a non-invasive detection method and biomarker are still needed. To this end, detection of UC from a urine sample by droplet digital PCR (ddPCR) has been made possible through the use of assessing copy number (CN) alterations (offered as BRAF Complete by LABOKLIN GmbH & Co KG in Europe and CADET^®^ BRAF-PLUS by Antech in the USA (Fountain Valley, CA, USA)), which can be helpful in dogs whose UC does not have a *BRAF* p.V595E mutation. Specifically, CN gains on chromosomes 13 and 36 and losses on chromosome 19 were evident in >75% of cases of canine UC, with >93% showing two or more of these CN alterations [45]. These alterations were absent in urine samples from dogs with urinary tract infections, cystitis, or benign bladder polyps [46].

In a recent investigation of over 140 canine UC and PCs, it was confirmed that an anti-human BRAF p.V600E-specific antibody could effectively and reliably identify the mutant BRAF V595E protein by immunohistochemistry [47]. Sixty percent of the examined carcinomas were found to be positive for the BRAF mutation by immunohistochemistry (IHC; which was confirmed by ddPCR), and all benign prostate and bladder tissues lacked the mutation. Thus, with the availability of tissue microarrays and IHC, large-scale screening of canine cancers by IHC for the presence of BRAF mutations has now become feasible.

**Figure 6:**
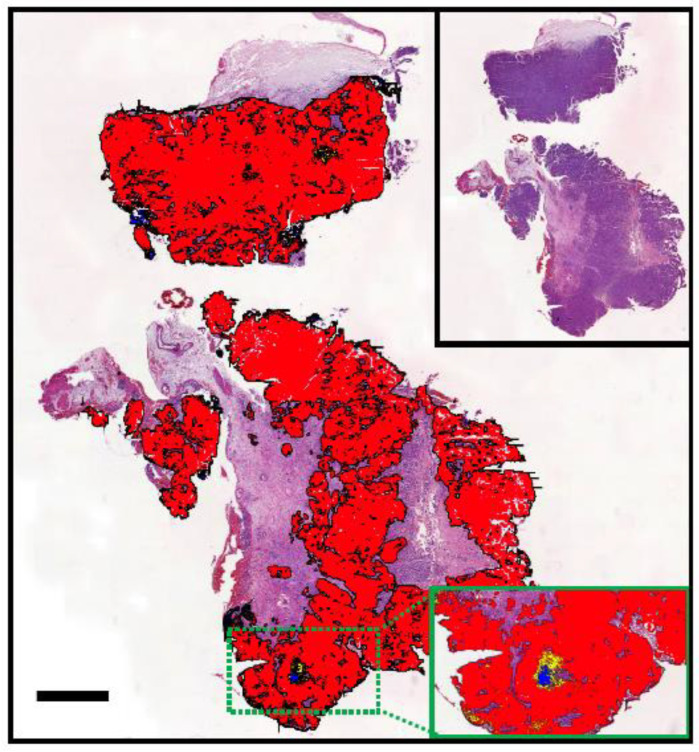
Bladder urothelial carcinoma of a 12-year-old West Highland White terrier tested positive for the *BRAF* mutation by PCR, which was accurately predicted by AI histology. Labelling: red: BRAF positive; blue: BRAF negative; yellow: uncertain (HE stain, bar = 1 mm).

A rapidly evolving technique that has been authorised for clinical purposes in human medicine since 2021 [48] is the use of artificial intelligence (AI) to detect cancer or corresponding molecular alterations from digitised tissue sections (reviewed in [49,50]). In veterinary science, this method was recently shown for the first time in a study of canine bladder tumours to detect the *BRAF* p.V595E mutation through commercial AI histology software (Visiopharm 2022.11, Hørsholm, Denmark) [51]. On whole slide images of urinary bladder UCs stained with haematoxylin and eosin, this specific mutation was predicted to be either present or absent for each pixel and overall (Figure 6). With a specificity of 63% and sensitivity of 58% (which increased to 89% sensitivity when small or poor-quality tissue sections were excluded), these results highlight the potential of AI in predicting molecular alterations in routine histology sections [51]. However, before this and similar tools can be implemented into veterinary diagnostics, the test performance must first be improved and thoroughly validated. Key aspects for increasing test accuracy include using large and balanced training sets with tissue sections of high sample, staining, and scanning quality.

#### 4.1.4. Use as a Prognostic Biomarker in Canines

There are two studies on the prognostic relevance of the *BRAF* p. V595E mutation in dogs with cancer, particularly UC. A study of 29 dogs with UC of the urinary bladder or urethra found no significant difference in survival times based on the mutational status of *BRAF*. However, they did find a trend for longer mean survival times (MST) for dogs with *BRAF* p.V595E (*n* = 16) versus *BRAF* wildtype tumours (*n* = 13; 11 months versus 5 months, *p* = 0.05) [52]. A more extensive study of 79 dogs with UC of the urinary bladder or urethra found a trend towards shorter MST in dogs with *BRAF* p.V595E versus *BRAF* wildtype tumours (214 versus 359 days, *p* = 0.055) [33]. The differing results between the studies may be due to the different treatment regimens and patient selection criteria used.

Furthermore, the *BRAF* p.V595E status may be used for monitoring disease progression. Using allele-specific real-time PCR of *BRAF* p.V595E circulating tumour DNA in the plasma of dogs with urinary bladder UC (*n* = 15), one study found that the amount of *BRAF* p.V595E ctDNA increased with disease progression (tumour growth, metastasis) and decreased when the tumour reduced in size in response to treatment [53]. Similarly, in a case report of a dog with unresectable metastatic urinary bladder UC undergoing RAF-inhibitor treatment (Sorafenib), longitudinal measurement of *BRAF* p.V595E ctDNA to monitor the treatment response found that levels followed the clinical course of the patient [54]. Specifically, peak *BRAF* p.V595E ctDNA levels were seen during increased urethral wall thickness and worsening periods of dysuria, and *BRAF* p.V595E ctDNA levels were barely able to be detected when the urethral wall thickness significantly decreased and the dysuria resolved [54]. Of course, further validation with larger patient numbers is required to determine the suitability of *BRAF* p.V595E ctDNA as a prognostic marker and/or progression marker for canine cancer.

#### 4.1.5. Use as a Predictive Biomarker in Canines

The evolutionary conservation of the BRAF p.V600E mutation highlights the importance of activating the MAPK pathway in cancer. It offers an opportunity for molecular diagnostics and targeted therapeutics for dogs with BRAF-mutated tumours, as it has for humans with *BRAF* p.V600E tumours.

One study found that survival of the dogs with *BRAF* p.V595E urinary bladder tumours was significantly dependent upon the treatment regimen, and those receiving adjuvant metronomic chlorambucil after mitoxantrone showed greater than double the MST compared to patients receiving mitoxantrone alone (588 versus 216 days, *p* = 0.030). In comparison, no significant differences in the MSTs were seen amongst the different treatment regimes in dogs with *BRAF* wild-type tumours (*p* = 0.069) [33].

*In vitro* studies using canine BRAF p.V595E UC cell lines have found mixed results regarding sensitivity to BRAFi. One study found that canine BRAF p.V595E UC cell lines were significantly less sensitive than human BRAF p.V600E cell lines to the mutant BRAF inhibitor vemurafenib (PLX4032), as determined by cell proliferation assays [55]. Instead, they found a similar level of sensitivity between the cell lines when using a second-generation BRAF inhibitor, PLX7904 [55], which belongs to the ‘paradox breaker’ class of BRAFi, as they do not exhibit the paradoxical ERK activation in *BRAF* wild-type cells seen in the traditional BRAFi, such as vemurafenib [56]. Another study found that canine BRAF p.V595E UC cell lines were sensitive to the mutant BRAF inhibitor vemurafenib (PLX4032), showing reduced cell viability and increased apoptosis [57]. However, they were much more sensitive to sorafenib (BAY 43-9006) [57], a multi-kinase inhibitor that targets numerous serine/threonine and tyrosine kinases (BRAF, RAF1, PDGFR, VEGFR1-3, KIT, FLT3, FGFR1, and RET).

A tolerability study performed on dogs with cancer (including three with UC) found that sorafenib was well tolerated, with no evidence of adverse effects related to the drug [58]. In addition, clinical activity was suggested in these three dogs with UC, as no disease progression was noted for >4 weeks [58]. A Phase I/II clinical trial of vemurafenib in dogs with *BRAF* mutant UC determined that the observed safety, anti-tumour activity, and cutaneous pharmacodynamic effects of vemurafenib (specifically the development of cutaneous squamous cell carcinoma and papillomas) closely mimicked those reported in humans [59]. Thus, the *BRAF* mutation status in canine UC and PC offers a new therapeutic strategy for individualised treatment. However, it should be noted that the treatment is very expensive.

#### 4.1.6. BRAF Alterations in Feline Cancer

Tumours in cats have not been as extensively studied as those in dogs, and in particular, there are comparatively few investigations into the genetics of feline tumours [60]. Only two studies have assessed the presence of *BRAF* mutations in feline tumours. In the first study, sequencing of the feline genomic region orthologous to the human *BRAF* p.V600E mutation in feline ocular melanomas (*n* = 10) did not find the presence of any mutations, with gene expression analysis showing reduced *BRAF* expression in the tumours relative to normal tissue [61]. However, the same group later used IHC and found increased expression of BRAF protein in feline ocular melanomas [62]. Thus, further research is needed into the role of BRAF in feline ocular melanomas.

In the second study, whole-exome sequencing of feline urinary bladder UC (*n* = 23) was performed, and in contrast to canine urinary bladder UC, no mutations in the *BRAF* gene were present [36]. Given the paucity of knowledge of mutations in *BRAF* in feline tumours, it is not used as a molecular biomarker in feline oncology.

### 4.2. KIT

**Figure 7:**
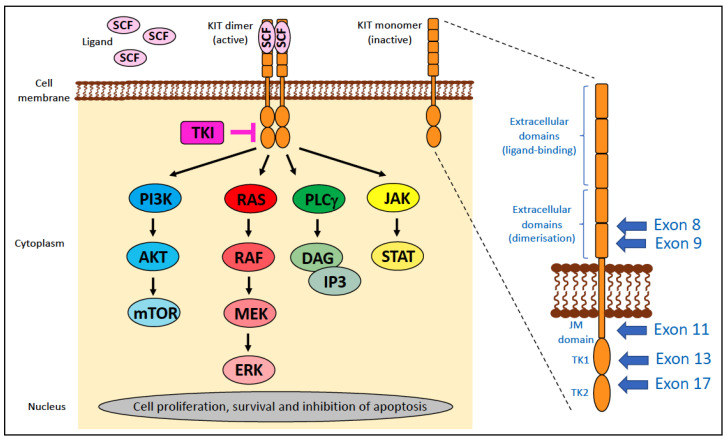
KIT structure and signalling. A schematic overview of the receptor tyrosine kinase KIT, its activation by the stem cell factor (SCF), and some of the main signal transduction pathways leading to KIT-induced cell proliferation, survival, and inhibition of apoptosis. Abbreviations: JM, juxtamembrane; TK, tyrosine kinase; TKI, tyrosine kinase inhibitor.

The proto-oncogene KIT, also known as c-KIT, CD177, or stem cell factor (SCF) receptor, is a receptor tyrosine kinase (RTK) encoded by the *KIT* gene. This cell-surface receptor consists of extracellular ligand-binding domains that interact with the SCF ligand (exons 1–9), a single transmembrane segment (exon 10), a juxtamembrane (JM) domain involved in signal transduction (exons 11–12), and a cytoplasmic tyrosine kinase (TK) domain, which is split into the ATP-binding lobes (TK1; exon 13) and the phosphotransferase lobes (TK2; exon 17; Figure 7).

When KIT is not bound to its ligand, it exists in the cell membrane as a monomer in a cis-autoinhibited state due to the JM domain inserting between the TK1 and TK2 domains, thus sterically blocking the “activation loop” within the catalytic cleft (Figure 7). The binding of a SCF dimer to the extracellular domains forms a bridge between two adjacent KIT molecules, resulting in homodimerisation, which leads to the trans-autophosphorylation of selected tyrosine residues in a specific order. The net result is a conformational change that allows access of ATP to the active site and activation of KIT (reviewed in [63]).

Signal transduction pathways downstream of activated KIT include the mitogen-activated protein kinase/EC signal-regulated kinase (MAPK/ERK), phosphatidylinositol 3-kinase/protein kinase B (PI3K/AKT), Janus kinase/signal transducer and activator of transcription (JAK/STAT), and phospholipase C-γ (PLCγ) pathways (Figure 7) [64]. KIT is crucial for stem cell maintenance, haematopoiesis, gametogenesis, melanogenesis, and in mast cell activities. Dysregulated KIT function promotes tumourigenesis and progression various cancer types through its activation or inappropriate expression.

Gain-of-function (GOF) mutations within the *KIT* gene in both humans and dogs and cats can be classified into two types based on their location within the gene [65]:

- ‘regulatory’ type—affects portions of the molecule that regulate kinase activity, such as releasing the inhibitory regulation of ligand-unoccupied c-Kit, resulting in constitutive activation. These include mutations in exons 8, 9, and 11.
- ‘enzymatic pocket’ type—affects the activation loop at the entrance to the enzymatic ‘pocket’. These include mutations in exons 13–21, typically exon 17.

#### 4.2.1. KIT Alterations in Human Cancer

*KIT* mutations have been reported in a wide range of human tumour types, with GOF mutations in exons 8, 9, 11, 12, and 17 being the most common. However, the location of the mutations varies with tumour type. For example, systemic mastocytosis is virtually always associated with activating mutations in *KIT* (>90% cases), specifically the *KIT* p.D816V mutation in exon 17 [66]. Activating mutations in *KIT* are the most common mutations found in gastrointestinal tumours (GISTs; 75–80% of cases [67]) and occur predominantly in exon 11 [68]. In contrast, the activating *KIT* mutations frequently found in acute myelocytic leukaemia (AML; 60–80% of cases) predominantly occur in exons 8 and 17 [69]. Similarly, *KIT* mutations in malignant melanoma have been identified predominantly in exon 11 [70,71]. The most common *KIT* alterations in the germ cell tumour, seminoma, are activating mutations in exons 11 and 17 (10–40% of cases) [66].

KIT is an RTK and thus can be targeted by tyrosine kinase inhibitors (TKIs). TKIs bind directly to the ATP-binding site of tyrosine kinases and block autophosphorylation, thereby preventing activation caused by regulatory-type mutations and inhibiting tumour growth [72] (Figure 7). Imatinib is the paradigm of class III TKIs; with outstanding efficacy and relative safety, it is approved for the treatment of chronic myeloid leukaemia, GISTs, and certain types of haematological malignancies carrying *KIT* mutations [73]. However, the use of imatinib over time is associated with the occurrence of secondary resistance [74]. There are a range of mechanisms of resistance to TKIs, the most commonly reported being point mutations in the kinase domains [75,76] with others including gene amplification and/or overexpression, constitutive activation of the downstream signal transduction of RTKs, overproduction of P-glycoprotein, and inhibition of the transporter responsible for the uptake of imatinib into cells [77,78].

#### 4.2.2. KIT Alterations in Canine Cancer

**Figure 8:**
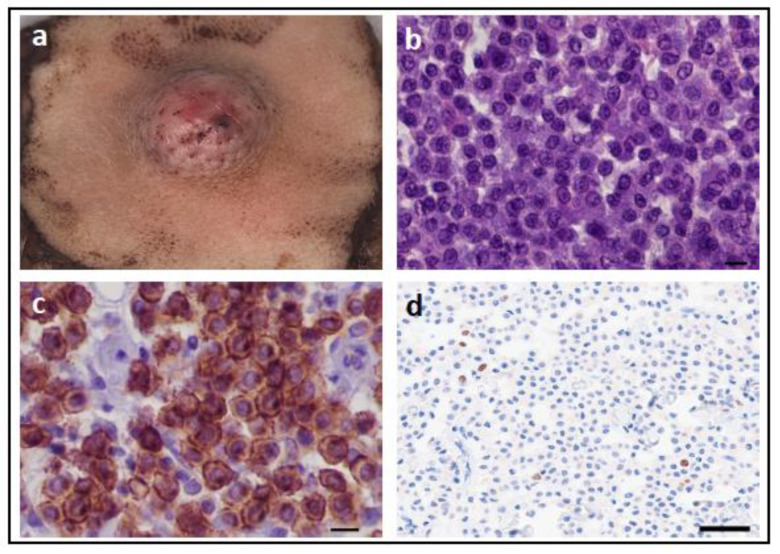
Canine cutaneous mast cell tumour from the sternum of a 7-year-old male Labrador. (**a**) Macroscopically, the nodule is 2 cm in diameter and was excised with wide margins after cytological diagnosis of a mast cell tumour (formalin-fixed sample). (**b**) Histology showed a well-differentiated mast cell tumour (Kiupel low-grade, Patnaik grade I; HE stain, bar = 10 µm). (**c**) Immunohistochemistry identified a normal perimembranous expression pattern of KIT (KIT IHC stain, bar = 10 µm). (**d**) Ki67 immunohistochemistry with a low number of tumour cells with positive nuclear (brown) labelling (Ki67 IHC, bar = 50 µm).

Mutations in *KIT* have been the most extensively studied in canine cutaneous mast cell tumours (MCTs; Figure 8). Between 5–40% of canine cutaneous MCTs carry mutations in *KIT* [79,80,81,82,83], and most of these are regulatory-type GOF mutations in exons 8, 9, 11, or 17 [79,81,84]. The most common mutations are internal tandem duplications (ITDs) in exon 11, followed by ITDs in exon 8, and then point mutations or small insertions or deletions in exon 8, 9, 11, and 17 [79,80,81,84,85,86].

**Figure 9:**
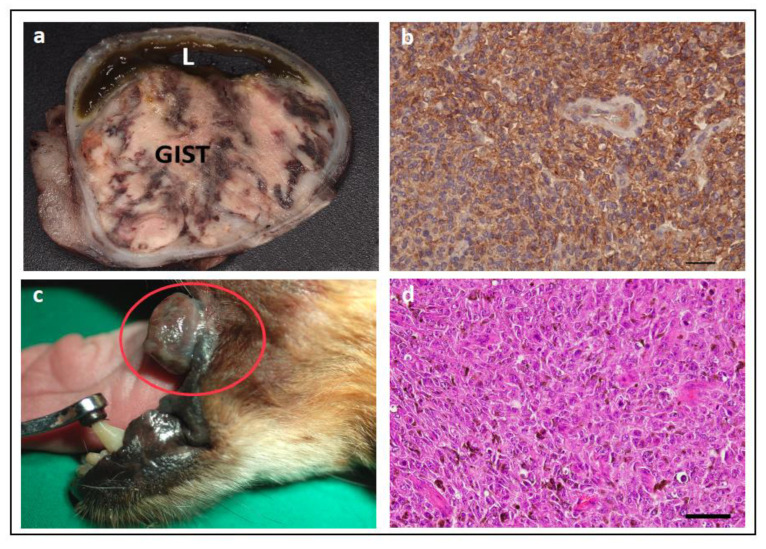
Gastrointestinal stromal tumour and oral melanoma. (**a**,**b**) Gastrointestinal stromal tumour (GIST) at the ileocaecal region from a 14-year-old castrated male Labrador. (**a**) Macroscopically, a 5 cm white-brown nodule derived from the intestinal wall was identified, which compressed the intestinal lumen (L). (**b**) Immunohistochemically, an intense KIT expression confirmed the cellular origin and differentiated the neoplasm from leiomyoma/leiomyosarcoma and fibrosarcoma (KIT IHC, bar = 25 µm). (**c**,**d**) Oral melanoma from a 16-year-old neutered female dachshund. (**c**) Macroscopically, a 1.5 cm brown nodule at the corner of the mouth was identified. Photo courtesy of Dres. Staudacher (AniCura, Aachen, Germany). (**d**) Histologically, the tumour cells are pleomorphic and contain variable amounts of cytoplasmic dark brown pigment (melanin; HE stain, bar = 50 µm).

The presence of *KIT* mutations has been assessed in other canine tumour types, specifically GISTs and oral melanomas (Figure 9). Exon 11 mutations of *KIT* have been reported to be a frequent occurrence in canine GISTs, ranging from 33–74% and including deletions, ITDs, and point mutations [87,88,89,90,91,92]. In contrast, mutations in other exons have only been reported in one study (in exon 9) [93].

The mutation status of *KIT* in canine melanomas has had contrasting reports. One study found no mutations in exon 11 (*n* = 17 cases) [94], while another reported exon 11 mutations in 5/49 (10%) cases [95]. A case report also described a melanoma with a mutation in exon 11 (a 9 bp deletion) [96]. Another study found no mutations in exons 13, 17, and 18 [97]. WES of canine oral melanomas (*n* = 65) found no mutations in *KIT* [98]. Yet, next-generation sequencing (NGS) of *KIT* found mutations with predicted deleterious effects in 8/27 (30%) canine oral melanoma cases (although none were in exon 11) [99]. The same study found *KIT* mutations in only 1/12 (8%) cases of canine histologically well-differentiated oral melanocytic neoplasms [99]. In canine digital melanoma, exon 11 mutations in *KIT* were found in 16/70 cases (23%) [100].

#### 4.2.3. Use as a Diagnostic Biomarker in Canine Cancer

The presence or location of mutations in *KIT* is not used as a diagnostic biomarker for canine MCT, GIST, or melanoma, with the gold standard being histopathological diagnosis. Rather, KIT is routinely assessed by IHC as a diagnostic marker to distinguish between different cancer types, such as MCT (KIT IHC positive) versus lymphoma (KIT IHC negative), or GIST (KIT IHC positive) versus fibrosarcoma (KIT IHC negative).

#### 4.2.4. Use as a Prognostic Biomarker in Canine Cancer

**Figure 10:**
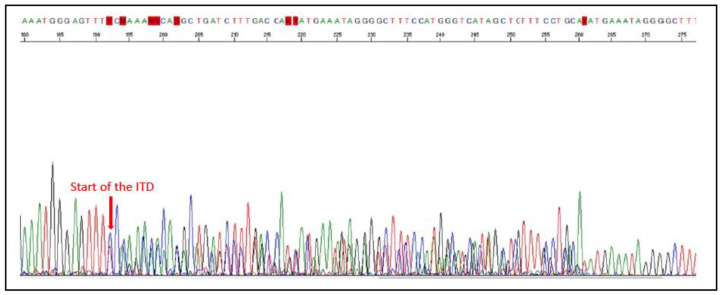
Electropherogram of a *KIT* exon 11 containing an internal tandem repeat (ITD). The DNA sample is from the mast cell tumour of an 8-year-old male Scottish Terrier.

ITDs in exon 11 of *KIT* in canine cutaneous MCTs are associated with aberrant KIT protein localisation, increased cellular proliferation, and a higher histological grade. In addition, MCTs with these mutations have an increased risk of recurrence and metastasis, which is associated with decreased survival times and a high risk of MCT-related mortality [81,85,86,101,102,103,104]. In contrast, exon 8 mutations of *KIT* in canine cutaneous MCTs are associated with a lower histologic grade and proliferation activity, less often aberrant KIT localisation, and are not associated with poor prognosis (MCTs with mutations in exon 8 of *KIT* were associated with longer overall survival (OS) times than those without mutations in exon 8 or 11) [104,105]. It has been proposed that combining histologic grading, together with analyses of cell proliferation indices (mitotic figures, Ki67, AgNORs), KIT immunohistochemistry, and detection of *KIT* mutations, provides the most detailed prognostic assessment for canine cutaneous MCT (Figure 10) [105,106,107].

One study found that four cases of dogs with GISTs that developed abdominal metastases all showed the presence of *KIT* mutations, suggesting a potential association of *KIT* mutation with more aggressive biological behaviour [92]. Similarly, one study found that canine melanomas carrying the mutations in exon 11 of *KIT* significantly correlated with disease recurrence (*p* = 0.05) [95]. Thus, more studies are warranted to assess the prognostic potential of *KIT* mutations in canine GISTs and melanomas.

#### 4.2.5. Use as a Predictive Biomarker in Canine Cancer

*KIT* alterations are a predictive biomarker for dogs with cutaneous MCTs. Two RTK inhibitors are currently in use for the treatment of recurrent, non-resectable grade II and III canine MCTs, with the latter requiring confirmation of the presence of *KIT* mutations in the tumour [108,109]. Toceranib (Palladia™) has registered approval with the United States Food and Drug Administration (FDA) and the European Medicines Agency (EMA), and masitinib (Masivet™) has registered approval with the EMA (note it is not registered in the United States). However, it is worth noting that although both target KIT, they also target other RTKs. Toceranib also inhibits VEGFR2 and PDGFR, and masitinib also targets PDGFR, FGFR3, and FAK [108,110,111].

Canine MCTs carrying *KIT* mutations generally respond well to TKIs. For example, a Phase I trial of toceranib in dogs with a range of malignancies found the highest response rate (significant shrinkage or stabilisation of disease) amongst the dogs with MCTs, particularly those showing *KIT* mutations [112]. A randomised, Phase 3 clinical trial of masitinib demonstrated it was effective at delaying tumour progression in dogs with recurrent or non-resectable grade II-III non-metastatic MCT (increased OS from 75 to 253 days), with the presence of *KIT* mutations showing an improved response [113]. A long-term follow-up study demonstrated increased survival rates at 12 and 24 months (40% vs. 15% at 24 months for masitinib vs. placebo) [114]. Other studies have also reported that dogs with cutaneous MCTs possessing *KIT* mutations had a significantly longer time to progression when treated with imatinib [108,115]. However, it is essential to note that some studies have not found *KIT* mutation status to be predictive. For example, a study of high-risk canine cutaneous MCTs (specifically those presenting with macroscopic cutaneous MCTs at disease stage II or III) found that Ki67, KIT immunolabelling, and the exon 11 mutation of *KIT* did not provide predictive information regarding response to TKIs in this population [116]. Similarly, other studies have found that the *KIT* mutation status does not predict treatment response to imatinib [113,117] or toceranib [118].

Case reports describing the use of imatinib in the setting of dogs with metastatic MCTs (of either cutaneous [115] or intestinal [119] origin) harbouring *KIT* mutations have described an initial marked decrease in tumour size. However, the clinical response tends to be short-lasting. Indeed, as in humans, resistance to TKI therapeutics has been reported in canines with MCTs due to the presence of additional mutations in *KIT* [120,121,122,123]. As such, knowledge of all mutations within *KIT* is essential as it influences whether the MCT will be responsive or resistant to TKI therapy and, as such, is necessary for the implementation of a stratified and more effective medical approach [123,124].

A case report of a canine with non-resectable GIST with exon 11 mutations in *KIT* showed partial remission with imatinib treatment [90]. Similarly, successful use of toceranib has also been reported in dogs with metastasized and/or non-resectable GIST [93,125,126,127]. However, the success of toceranib does not always correlate with the presence of *KIT* mutations [125,127], and in some cases, the *KIT* mutation status has not been assessed [126].

A case report of a canine with metastatic oral melanoma carrying an exon 11 mutation in *KIT* that was treated with toceranib reported initial improvement of tumour-associated clinical signs and reduced size of the tumour and metastatic lymph node; however, therapy was terminated on day 43 due to disease progression, and the dog died on day 54 [96]. Thus, at present, there is insufficient data to confirm if *KIT* mutations are a predictive biomarker for canine GISTs and MMs.

#### 4.2.6. KIT Mutations in Feline Cancer

**Figure 11:**
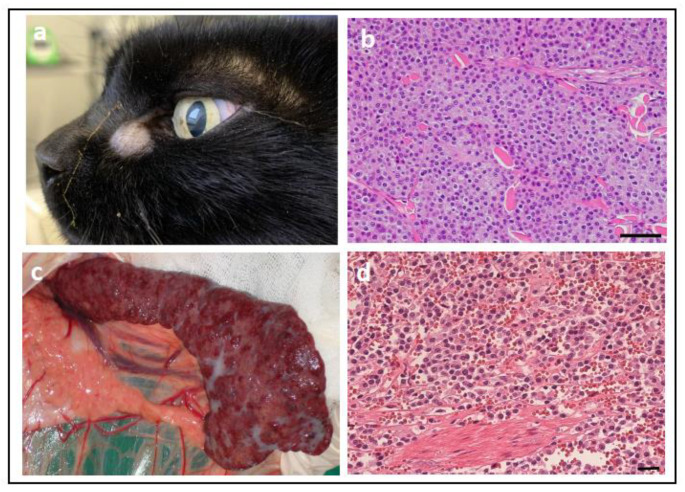
Presentations of feline mast cell tumours (MCT). (**a**) Macroscopically, a cutaneous MCT on a 14-year-old male castrated domestic short-hair cat. Photo courtesy of Dr. Miriam Stöckli (Vetsuisse Faculty, University of Bern). (**b**) Histologically, neoplastic mast cells are moderately pleomorphic (HE stain, bar = 50 µm). (**c**) Macroscopically, a moderately enlarged (14.0 × 2.0–7.0 × 1.0 cm) spleen with multifocal fibrosis of the capsule and multinodular surface was removed from the abdominal cavity of an 8-year-old castrated male domestic short-haired cat. Photo courtesy of Dres. Staudacher (AniCura, Aachen, Germany). (**d**) Histologically, diffuse infiltration of the splenic parenchyma with pleomorphic mast cells (case from Figure (**c**); HE stain, bar = 25 µm).

A study of 62 cats with either cutaneous, splenic, or widespread MCTs (Figure 11) identified *KIT* mutations in 67% of cases, with the vast majority of them being in exons 8 and 9 [128]. *KIT* mutations have been observed in 56% of feline cutaneous MCTs, predominantly occurring in exon 9 (71%), followed by exon 8 (19%) and exon 11 (10%) [129]. Conversely, *KIT* mutations have been found in 65% of feline splenic MCTs, occurring predominantly in exon 8 (79%) followed by exon 9 (21%), with no mutations observed in exons 11, 12, and 17 [130,131]. A case report of systemic mastocytosis and mastocytemia in a cat identified an ITD in exon 8 of *KIT* [132]. On the other hand, no *KIT* mutations have been found in feline intestinal MCTs [133].

#### 4.2.7. Use as a Diagnostic Biomarker in Feline Cancer

The presence of mutations in *KIT* is not used as a diagnostic biomarker for any feline cancer. Rather, as mentioned for canines (Section 4.2.3), KIT is routinely assessed by the IHC as a diagnostic marker to distinguish between different cancer types.

#### 4.2.8. Use as a Prognostic Biomarker in Feline Cancer

A study of feline cutaneous MCTs (*n* = 34) found that *KIT* mutations were not significantly related to protein expression and did not influence prognosis [129]. Another study of feline cutaneous MCTs (*n* = 20) found no significant association between the KIT mutation status and tumour histological grade or mitotic index [134]. Similarly, a study of feline splenic MCTs (*n* = 20) found no correlation between *KIT* mutations and tumour differentiation, mitotic activity, or survival [131].

#### 4.2.9. Use as a Predictive Biomarker in Feline Cancer

While toceranib and masitinib are licensed for use in dogs, no TKI is yet approved for cats [135]. However, a retrospective study of cats with cutaneous, visceral, or gastrointestinal MCTs treated with toceranib found clinical benefit in 40/50 (80%) cases [136]. However, it is important to note in this study that the *KIT* mutation status of the tumours was not assessed, and as such, it is not possible to conclude whether the mutation had a predictive effect. An in vitro study examining the effect of TKIs (imatinib, midostaurin, nilotinib, and dasatinib) on spleen-derived neoplastic mast cells from three cats with systemic mastocytosis and carrying mutations in exon 8 of *KIT* found a dose-dependent growth-inhibitory effect associated with morphologic signs of apoptosis [137]. A case report of feline systemic mastocytosis and mastocytemia with a mutation in exon 8 of *KIT* detailed a favourable response to treatment with imatinib, with the tumour masses undetectable after 5 weeks of treatment and the number of mast cells in the peripheral blood markedly reduced [132]. The same group demonstrated that 7/8 cats with MCTs (cutaneous, splenic, or widespread) that carried mutations in exons 8 or 9 of *KIT* showed a beneficial response to imatinib [128]. More recently, a case report of a feline with splenic and cutaneous MCTs that carried exon 8 mutations of *KIT* was treated with toceranib and showed complete remission until a recurrence on day 117. Despite removing the lesions, the cat died on day 191 [138]. Subsequent examination of the splenic MCT that was removed revealed additional mutations in exons 9, 10, and 18, suggesting heterogeneity among the population of tumour cells exists in MCTs and may have contributed to the re-growth of the tumour [138]. However, the TKI might have influenced the development of this heterogeneity. Thus, although encouraging, additional studies are warranted to thoroughly assess the predictive role of *KIT* mutations in feline MCT.

To date, there is only one report of a feline with GIST being treated with TKIs (imatinib, followed by toceranib, due to the owner’s desire to avoid surgical treatment); the tumour was unresponsive. However, subsequent analysis of the tumour did not find any mutations in *KIT* or *PDGFR*, which may account for the poor response [139].

### 4.3. PCR for Antigen Receptor Rearrangement in Dogs and Cats

**Figure 12:**
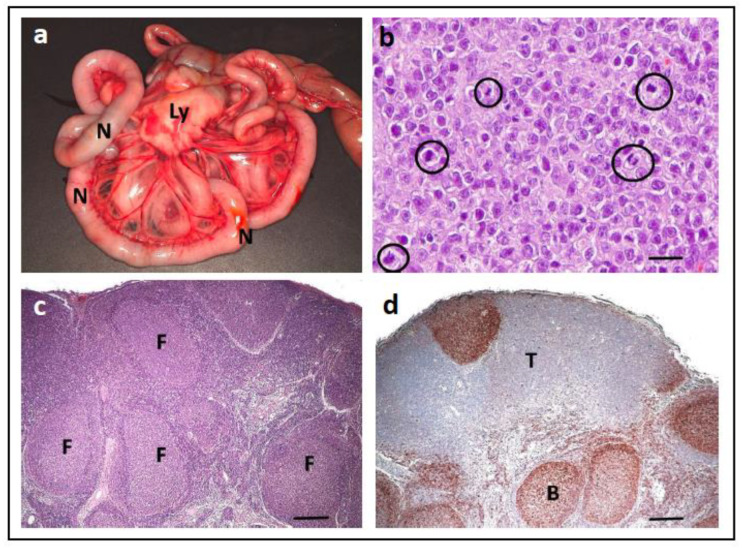
Alimentary lymphoma in a cat. (**a**) Macroscopically, a markedly enlarged mesenteric lymph node (Ly) and multiple nodules in the intestinal wall (N) due to an alimentary lymphoma in a 13-year-old domestic short-hair cat. (**b**) Histology showed a large-cell lymphoma with multiple mitoses (circle; HE stain, bar = 25 µm). (**c**) Histological picture from a marked proliferation (most likely hyperplasia; confirmed by PARR) of the follicles (F) in a lymph node of a 1-year-old mixed-breed dog (HE stain, bar = 250 µm). (**d**) Immunohistochemistry (CD79a) in this lymph node reflects the normal distribution of B-lymphocytes in follicles but gives no information about the clonality of the proliferation of cells (CD79a IHC, bar = 250 µm). T: T lymphocytes and B: B lymphocytes.

PCR for antigen receptor rearrangement (PARR) is a molecular test with the results used as a diagnostic biomarker for lymphoid malignancies in dogs and cats, specifically B- and T-cell lymphoma (Figure 12a,b). Specifically, as histopathologic discrimination can be difficult in some cases, it is used in cancer diagnostics to differentiate lymphoma from benign processes. This is especially relevant for marked reactive lymphofollicular proliferation (Figure 12c,d), which can be challenging to differentiate from follicular lymphoma (e.g., mantle cell lymphoma). If sufficient numbers of relevant cells are available, PARR can be performed from various sample types, such as cytological smears, FFPE tissues, effusion fluids, or cerebrospinal fluid.

PARR does not detect somatic mutations, but rather identifies naturally occurring somatic rearrangements of antigen receptor genes that occur during lymphocyte development/maturation [140,141]. Lymphocytes undergo a process of antigen receptor gene rearrangement during their development; B-cells rearrange the immunoglobulin heavy chain (IgH) genes to allow for every B-cell to have a unique antibody, and T-cells rearrange the T cell receptor (TCR) genes to have a unique TCR on each T-cell. Upon activation by a specific antigen, T-cells undergo clonal proliferation/amplification; thus, in normal lymphoid tissues, this process results in a polyclonal population with a wide variety of B- and T-cells, each with their own unique receptor sequences. In lymphomas, however, the malignant lymphoid cells arise from a single clone, specifically one B- or T-cell that proliferates unregulated. Thus, PARR is used as a molecular diagnostic biomarker to detect and analyse the clonality of the B- and/or T-cell populations of a sample [142,143,144,145,146].

**Figure 13:**
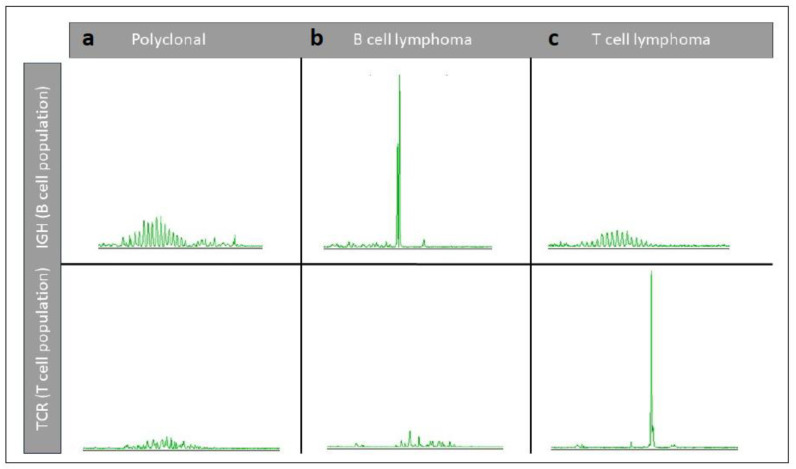
PARR-GeneScanning-based clonality test results from three cats. (**a**) A 4-year-old castrated female cat with a polyclonal B and T cell population. (**b**) A 10-year-old male cat with a B-cell lymphoma. (**c**) A 16-year-old castrated female cat with a T-cell lymphoma. *x*-axis: fluorescence intensity; *y*-axis: PCR product size.

PARR involves the use of PCR to selectively amplify the variable regions of antigen receptor genes, such as the various IgH or TCR genes. Using primers that target conserved regions flanking the variable regions of the IgH or TCR genes, PCR amplifies specifically the rearranged segments of the variable regions; the resulting DNA fragments are analysed to determine their size and pattern by various methods/techniques. In healthy samples, a great variety of PCR products resembling the broad spectrum of gene rearrangements can be detected (a ‘polyclonal’ population). In contrast, the presence of a single PCR product (a ‘monoclonal population’) is indicative of a clonal expansion, (i.e., the expansion of a single/clonal B or T cell, suggesting the presence of a lymphoma (Figure 13) [142,143,144,145,146].

It is important to note that PARR diagnostics has several challenges, such as false-negative results. False-negative results are when the dog or cat has lymphoma, but the PCR does not show the pattern of a monoclonal population. For PARR, detecting all potentially rearranged gene segments is crucial; if the primer pairs do not cover the particular gene rearrangement used by a neoplastic clone, the PARR result will be false-negative. However, with increased knowledge of the canine and feline genomes over the last few years, there has been significant improvement in the ability of PARR primers to cover both common and uncommon gene rearrangements [145,147]. However, it is important to note that, apart from issues in primer design, mutations in the primer binding sites due to naturally occurring hypermutations or chromosomal aberrations can also lead to false negative results [145].

False positive results are also a concern and can sometimes occur when there is monoclonal expansion (without the presence of neoplasia), such as clonal amplification due to antigenic stimulation as part of the normal immune response. However, in most cases, antigenic stimulation will affect several clones with different rearrangements, so PARR will still detect a polyclonal picture. Yet it is important to note that in some cases, a monoclonal pattern was seen by PARR after infection with *Leishmania infantum* [143,148] or *Ehrlichia canis* [141]. Furthermore, as canonical rearrangements in γδ T-cells were described as a cause of false positive results, their relevance in dogs and cats must be investigated [145].

Thus, it is recommended that the integration of clinical, morphologic, and immunophenotypic information is essential, and PARR should never be used as a standalone molecular diagnostic tool [145]. In addition, harmonisation/standardisation regarding the use of primer sets and the interpretation of results would be desirable, as is conducted in human medicine [142,144].

### 4.4. Multi-Gene Panels

The advent of next-generation sequencing (NGS) technology has revolutionised human medicine with the ability to obtain the DNA sequence of multiple genes in a single assay through the use of whole genome sequencing (WGS, i.e., all the genes in an individual), whole exome sequencing (WES, i.e., all the exonic regions of the genome), and targeted sequencing (TGS, a specific set of genes/genomic regions). NGS can assess somatic or germline alterations, such as SNVs, MNVs, indels, copy number changes, and chromosomal rearrangements (Figure 1). Thus, using NGS removes the constraint of *a priori* knowledge of which gene may be mutated (e.g., *BRAF* or *KIT*) and/or the type of genetic mutation/genomic alteration that may be present (e.g., a hotspot mutation or changes in chromosomal copy number). TGS is a relatively cheaper and faster alternative to WGS and WES. It uses custom-made ‘capture baits’ designed to complement the specific genes or genomic regions of interest. After hybridisation of the sample DNA with the baits, any unbound DNA is removed, and the bound DNA is then released and sequenced. Thus, only the ‘targeted’ regions of the genome are sequenced. As only a fraction of the genome is being sequenced, it can be conducted at much deeper levels (i.e., with more sequencing reads covering the genes/genomic areas of interest), allowing for the detection of lower-frequency variant alleles (mutations).

#### 4.4.1. Clinical Use of Multi-Gene Panels for Human Cancer Patients

Targeted sequencing has become widely used in human medicine for cancer patients, such as the MSK-IMPACT^®^ test offered by Memorial Sloan Kettering Cancer Center (MSKCC), which is the first laboratory-developed tumour profiling test to receive FDA approval. The test involves sampling the tumour and blood of the patient, from which DNA is extracted and sequenced across a panel of several hundred target genes (with new targets continually being added). The sequencing reads are then analysed through a custom bioinformatics pipeline that detects specific mutations, copy number alterations, and selected structural arrangements. The results are reported in the electronic medical record and also sent to an in-house database that facilitates automated clinical trial matching, as well as automatically uploading the sequencing data to the publicly-available cancer genomics cBioPortal database (https://www.cbioportal.org/ (accessed on 23 December 2023)) for data mining and interpretation. A study found that almost 37% of the first ~10,000 people to use the MSK-IMPACT^®^ test had at least one actionable (‘druggable’) mutation [21]. In addition, multi-gene panels use RNA extracted from FFPE tumour tissue to assess a patient’s gene expression profile (GEP). These are typically used for specific tumour types. For example, there are several commercially available panels for breast cancer patients that help clinicians with diagnostics, prognostics, and/or predictive responses to therapy, including Prosigna™ [149], MammaPrint^®^ [150], BluePrint^®^ [150], EndoPredict^®^ [151], and Oncotype DX^®^ [152].

It is important to note that genomically informed therapy requires knowledge of the functional impact of the genomic alteration, specifically its effect on expression levels and/or the functioning of the relevant protein(s). However, there are many genetic variants for which it is not known whether the ‘impact’ on the protein is detrimental and are termed ‘variants of unknown significance’ (VUS). For example, one study found that 48% of variant annotations provided to oncologists were VUS [153]. In an attempt to advise oncologists in such situations, a rule-based ‘actionability classification’ scheme has recently been proposed by the MD Anderson Precision Oncology Decision Support (PODS) team that categorises VUS as either “Unknown” or “Potentially” actionable, based on their location within functional domains and/or proximity to known oncogenic variants [154].

#### 4.4.2. Clinical Use of Multi-Gene Panels for Veterinary Cancer Patients

Numerous studies have used WGS, WES, and/or TGS to characterise the genetics of a large variety of canine tumours (reviewed in [8]); however, only three such studies have been performed on feline tumours to date, specifically TSG of cutaneous hemangiosarcoma [155], WGS of oral squamous cell carcinoma [156], and urinary bladder UC [36]. Reflecting this, several companies now perform TGS and analysis of canine patient samples, but they have yet to offer a service for feline samples.

The first multi-gene cancer panels for characterising somatic mutations in canine tumours are commercially available in the USA (such as the “SearchLight^®^ DNA” test from Anivive Lifesciences and the “FidoCure^®^” test from One Health Company, Cleveland, Ohio). The tissue sample for analysis can be FFPE tissue, fine needle aspirations, or urine/effusions, and the extracted DNA is used for TGS on a customised panel of 59–120 cancer-associated canine genes (specific details depend on which test is used). Analysis of the sequencing data allows identification of SNVs, indels, CNAs, and ITDs in *FLT3* and *KIT* (depending on the test). Typically, the identified molecular alterations are annotated to determine whether the scientific literature shows evidence of them being diagnostic, prognostic, and/or predictive biomarkers. The report also includes guidance on which targeted therapies are FDA-approved for humans with the same genetic alterations, and thus may benefit the dog.

Of course, the availability and cost of the drugs can vary from country to country, and the relative efficacy and potential side effects of these drugs on dogs are supported by varying degrees of evidence. The decision and responsibility for the medication listed in the report lie with the treating veterinarian. It is strongly recommended that a specialised veterinary oncologist be consulted, as would be the case in human medicine. Nevertheless, recent studies using these multi-gene cancer panels have demonstrated their relevance and clinical benefits [157]. Using the FidoCure^®^ platform, a study of 813 dogs with tumours spanning 53 cancer types found that almost 90% of the cases exhibited mutations with diagnostic, prognostic, or therapeutic implications [158]. A study of 671 dogs with tumours spanning 23 cancer types identified 18 mutational hotspots, of which eight were orthologous to human cancer hotspots and were clinically actionable [35]. A study of 1108 dogs with tumours who were enrolled in the ‘FidoCure^®^ Personalized Platform’ found that several human-targeted therapies were associated with a favourable prognosis when used to treat dogs with specific somatic molecular alterations [159]. Using the SearchLight^®^ DNA platform, a study of 69 dogs with ambiguous cancer diagnoses found that it provided diagnostic information in 37/69 (54%) cases and prognostic and/or therapeutic information in 22 of the remaining 32 cases (69%) [160]. In a separate study of 127 dogs with cancer, it was found that mutations in 6 genes were significantly associated with shorter progression-free survival (PFS; *CCND1*, *CCND3*, *SMARCB1*, *FANCG*, *CDKN2A/B*, and *MSH6*) [161]. In addition, the dogs that received targeted therapy before the first progression (*n* = 24) experienced significantly longer PFS than those that did not receive the treatment (*n* = 82; *p* = 0.01), and considerably improved PFS was also found in the dogs that received genomically-informed targeted treatment (*n* = 29) relative to those that received treatment (targeted or otherwise) that was not genomically-informed (*n* = 98, *p* = 0.05) [161].

#### 4.4.3. Use of Multi-Gene Panels for Cancer Screening in Dogs

As cancer is a leading cause of death in dogs and cats, it suggests that preventive benefit could be derived from the establishment of regular cancer screening programs to detect cancer in preclinical patients who are at a higher risk of the disease due to their age or breed, as early detection and treatment are the best ways to manage cancer in pets. However, while robust guidelines exist for cancer screening programs in human medicine [162], options for dogs and cats are limited to various veterinary professional organisations and academic institutions issuing prevention and screening recommendations, as no formal guidelines for early detection of cancer through regular screening programs exist in veterinary medicine. A recent study that analysed data from 3452 cancer-diagnosed dogs suggested that annual cancer screening starting 2 years before the median age at cancer diagnosis for dogs of similar breed or weight would be reasonable to consider, and thus it is recommended to start cancer screening for all dogs at 7 years of age (or 4 years of age for breeds with a lower median age at cancer diagnosis) in order to increase the likelihood of early detection) [163].

In 2022, the world’s first cfDNA-based, non-invasive test for canine cancer detection became commercially available in the USA (OncoK9^®^, PetDx). In this multi-cancer early detection (MCED) test, a 14–17 mL blood sample (“liquid biopsy”) is taken from the dog and used to isolate cfDNA (short fragments of DNA released into the blood during cell death) and genomic DNA (gDNA) for NGS, to identify proprietary cancer-associated genomic alterations and provide a “Cancer Signal Detected/Cancer Signal Not Detected” result. A cohort of 1100 dogs (presumed to be ‘cancer free’) was used to assess the clinical performance of this MCED test, termed the CANDiD (CANcer Detection in Dogs) study, and found that overall, the test demonstrated a 54.7% sensitivity and a 98.5% specificity [164]. A follow up study of 1500 blood samples from dogs reported that the relative observed sensitivity was 61.5% and the specificity was 97.5%, with a positive predictive value of 75.0% for screening patients and 97.7% for aid-in-diagnosis patients [165]. However, it is important to note that both the tumour type and size of the tumour can influence the probability of a positive cancer signal [165]. An update as of March 2024: it is not clear whether this test is still available as there are reports that PetDx has closed down service.

To-date, no analyses have been performed or recommendations have been made regarding cancer screening in cats.

## 5. Germline Alterations Currently Tested in the Veterinary Clinical Setting

Tumour-predisposing germline alterations tested in the veterinary clinic are used as screening biomarkers and are mainly performed on DNA extracted from whole blood or buccal swab samples. They are typically used in the context of breeding advice as a genetic tool to assist breeders in making decisions regarding breeding partners and allow them to reduce the incidence of cancer in the canine population. There are several tumour-predisposing germline alterations in dogs for which clinical tests are available. However, due to the paucity of genetic investigations into feline cancers, clinical tests have yet to become available to assess the presence of tumour-predisposing germline alterations in cats.

### 5.1. Canine Hereditary Multifocal Renal Cystadenocarcinoma and Nodular Dermatofibrosis

**Figure 14:**
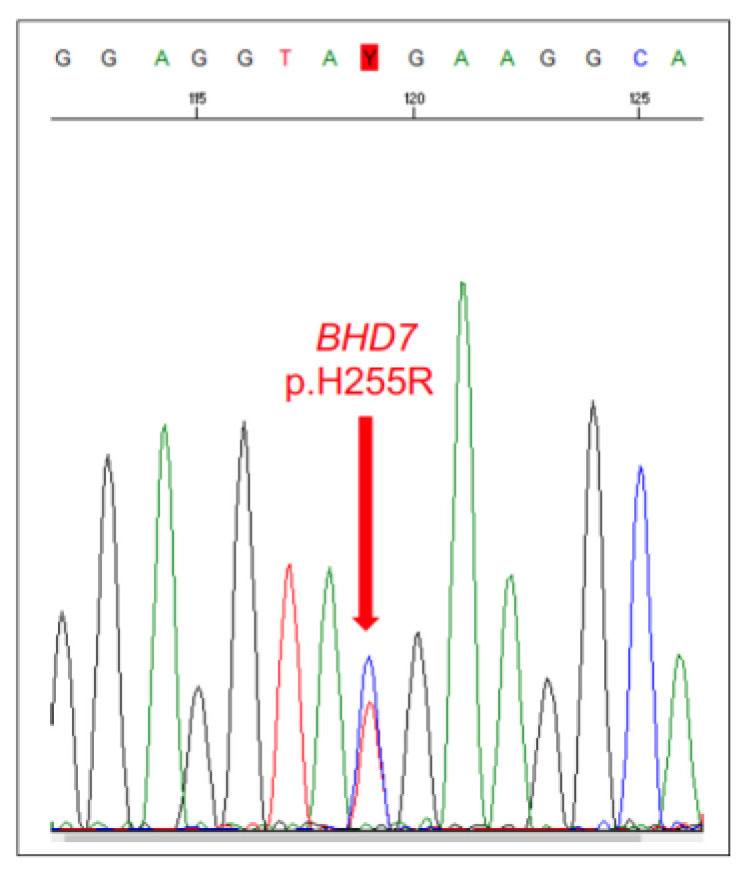
Electropherogram of the *BHD7* p.H255R exon 7 heterozygous variant that is causal for RCND. The sample is from an 8-year-old male German Shepherd.

Canine hereditary multifocal renal cystadenocarcinoma and nodular dermatofibrosis (RCND) is a rare inherited cancer syndrome observed in German Shepherd dogs (GSDs) [166]. This syndrome is characterised by bilateral, multifocal tumours in the kidneys, uterine leiomyomas, and nodules in the skin and subcutaneous tissue composed of dense collagen fibres. The disease typically presents in dogs > 5 years of age. However, tumours may not develop until much later, and death typically occurs at ~9 years of age due to kidney failure or metastatic disease. Investigations in the GSD population revealed that a missense mutation in exon 7 of the *BHD* gene (p.H255R) on chromosome 5 was associated with the disease and resulted in an amino acid change within a highly conserved region of the encoded protein, folliculin [167]. This mutation is present in the US and Norwegian GSD populations, having been separated over several generations. In addition, substantial evidence supports the notion that this *BHD* mutation may exert a homozygous lethal effect in the offspring. As such, the puppies would die early on in gestation [167]. The RCND test (offered by many companies) can be performed on blood or a buccal swab, and the extracted DNA is typically analysed by PCR to determine the presence of the *BHD* p.H255R mutation (Figure 14).

### 5.2. Familial Follicular Thyroid Carcinoma in German Longhaired Pointer Dogs

**Figure 15:**
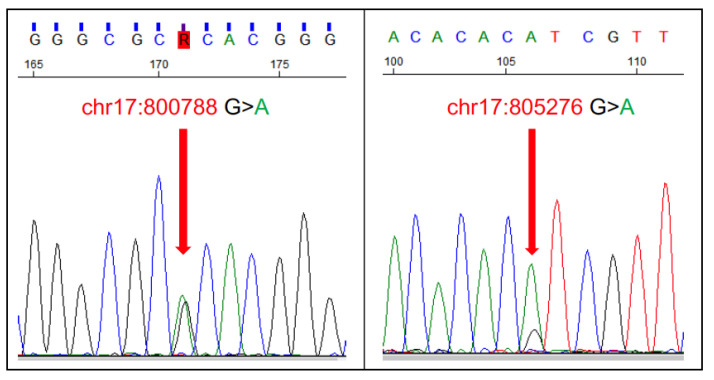
Electropherogram of a 5-year-old male German Longhaired Pointer dog that is heterozygous for *TPO* variants chr17:800788G>A and chr17:805276G>A. Note: variant chr17:805276G>A is the reverse complement (reverse strand) of the chr17:805276C>T variant.

Follicular thyroid carcinoma (FTC), also known as the ‘compact’ or ‘differentiated’ type, accounts for ~85% of thyroid carcinomas in dogs, and >50% of dogs with thyroid carcinoma have detectable metastasis at the time of diagnosis. Although FTC can arise spontaneously in the dog, there is also a familial form (FFTC) found in German Longhaired Pointers [168]. One study found the age of diagnosis between 4.5 and 13.5 years of age, with 76% of cases diagnosed before 10 years of age [168]. Through a comprehensive approach combining genome-wide association studies, analysis of runs of homozygosity, and whole-genome sequences, a specific region was identified spanning 0–5 Mb on chromosome 17, showing a significant association with the disease [169]. Subsequent whole-genome sequencing unveiled several mutations exhibiting a recessive inheritance pattern within this region. Notably, two deleterious mutations in the *TPO* gene were identified: chr17:800788G>A (*TPO* p.F686V) and chr17:805276C>T (*TPO* p.T845M), with genotyping results from a cohort of 186 dogs confirming a strong association between these genetic variants and the disease [169]. Dogs with homozygous affected genotypes exhibited notably higher relative risks than homozygous genotypes for the reference alleles [169]. Notably, a second study confirmed the familial nature of this cancer, as the affected dogs could be traced back to common ancestors and came from closely related Dutch pedigrees. There was strong evidence for a recessive mode of inheritance, although incomplete penetrance could not be ruled out completely [168]. The FFTC test (offered by many companies) can be performed on blood or a buccal swab, and the extracted DNA is typically analysed by PCR to determine the presence of the *TPO* p.F686V and p.T845M mutations (Figure 15).

### 5.3. Squamous Cell Carcinoma of the Digit in Black Poodles and Black Giant Schnauzers

Squamous cell carcinoma (SCC) is a malignant neoplasia arising from the epidermal layer and can show varying degrees of keratinocyte differentiation. SCC in dogs is most commonly seen in the skin, digits, and oral cavity, with each location displaying different biological behaviour and prognostic outcome [170,171]. SCC is the most commonly diagnosed tumour in the digits of canines, accounting for 47–63% of all the malignant neoplasms at this site [172,173]. SCC of the digit (DSCC) is a locally aggressive cancer that causes lytic bone lesions in ~80% of cases, with tumours often developing in multiple digits and ~19% of cases progressing to metastatic disease [174,175].

**Figure 16:**
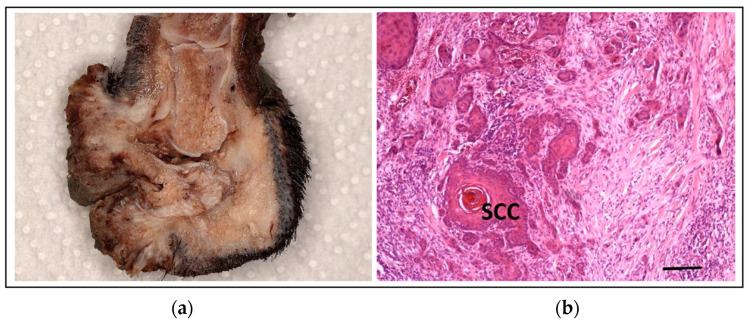
Squamous cell carcinoma (SCC) of the toe of a 7-year-old male Giant Schnauzer. (**a**) Macroscopic view of the longitudinal section through the toe, showing the loss of the claw and destruction of the phalanx by infiltrative tumour growth (formalin-fixed material). (**b**) Histologically, SCC is infiltrating the surrounding stroma (HE stain, bar = 50 µm).

Black-coated dogs, including Giant (GS) and Standard Schnauzers (SS), as well as standard poodles (STPOs), have an increased risk for SCCD (Figure 16). Interestingly, miniature schnauzers are only rarely affected [176], and only dark coat-coloured STPOs are at high risk, with light-coloured STPOs almost entirely unaffected. A genome-wide association study (GWAS) comparing dark-coated coloured STPOs with SCCD (*n* = 31) to unrelated dark-coat-coloured STPO controls (*n* = 34) identified a role for *KITLG* in SCCD susceptibility [177]. *KITLG* plays an essential role in melanogenesis (both developmentally and in the hair shaft) and has been identified as a pigmentation intensity locus in dogs. CNVs of *KITLG* have subsequently been shown to be responsible, among other things, for the different coat colours (pigment intensities) of dogs, with the median number of copies of *KITLG* varying from 2 and 8, depending on the breed and the coat colour [178,179]. A copy number variant (CNV) containing predicted enhancer elements for *KITLG* was found to be strongly associated with SCCD in STPOs, with ≥4 copies indicated as predisposing [177].

More recently, another study used ddPCR to identify that the significantly increased risk of developing SCCD in black-coated GS was associated with a CNV of *KITLG* >5.8 copies [176]. Furthermore, a significant correlation between *KITLG* copy number and the histological criteria of malignancy in SCCD has been shown [180].

**Figure 17:**
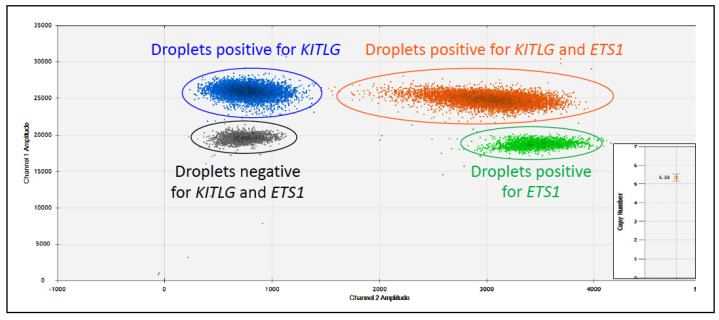
*KITLG* copy number assay. Raw data (2D plot) for the ddPCR of the *KITLG* copy number assay for a 3-year-old female Giant Schnauzer. *ETS1* is a reference gene used in the assay. Inset: analysis of the ddPCR data shows the dog has a *KITLG* copy number of 5.33.

The KITLG test (offered by LABOKLIN GmbH & Co KG, Bad Kissingen, Germany) can be performed on blood, and the extracted DNA is typically analysed by ddPCR to determine the *KITLG* copy number of the sample (Figure 17).

Interestingly, a study analysing the CNV of *KITLG* in blood samples from dogs with digital melanomas (*n* = 9) found 4–6 copies, with 4/5 of the dogs with a dark colour having >4 copies [100]. However, further analyses using a larger cohort and appropriate controls will be needed to validate that the CNV of *KITLG* could be involved in developing digital melanomas in dogs with dark-coloured coats.

### 5.4. Histiocytic Sarcoma in the Bernese Mountain Dog

**Figure 18:**
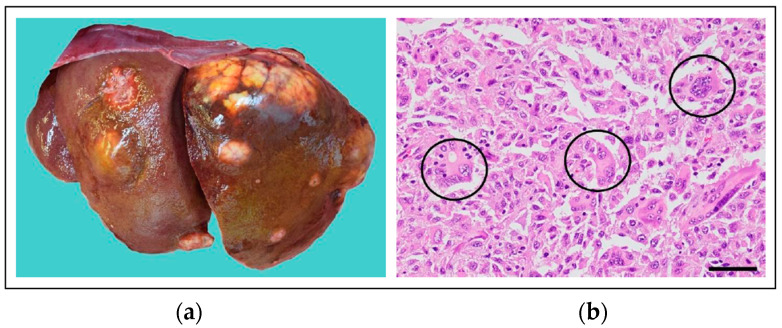
Histiocytic sarcoma in a 9-year-old neutered female Bernese Mountain Dog. (**a**) Macroscopically, multifocal to coalescing, tan, raised nodules are evident in the liver. (**b**) Histologically, tumour cells are highly pleomorphic, and multiple multinucleated cells are present (circles; HE stain, bar = 50 µm).

Histiocytic sarcoma (HS) is an aggressive tumour arising in dendritic cells and can present in two ways. The ‘localised’ form of HS typically occurs in the skin or subcutaneous tissue of the extremities, exhibits local invasiveness, and tends to metastasise to lymph nodes and blood vessels. In contrast, the ‘disseminated’ form of HS is a multisystemic disease, affecting various organs such as the spleen, liver, and lungs, and the progression of the disease is rapid, leading to a poor outcome [181]. Bernese Mountain dogs (BMDs) have a predisposition to developing HS, occurring in 15–25% of this breed [181,182,183] (Figure 18).

To identify the cancer-associated loci, both independent and combined GWAS studies were performed on genomic DNA from affected and unaffected BMDs belonging to two populations: North America (*n* = 240) and Europe (*n* = 95 from France and *n* = 140 from the Netherlands) [184]. Fine mapping and sequencing were then used to narrow the primary locus down to a single gene region, characterised by a single haplotype that spans *MTAP* and a segment of *CDKN2A* and was present on at least one chromosome (heterozygous) in 96% of BMDs, with 65% of the affected BMDs being homozygous BMDs [184].

Another study was able to confirm the finding of *CDKN2A/B* as a risk factor and expanded the risk markers to loci on canine chromosomes (CFA) 2, 5, 14, and 20. Additionally, the described markers on CFA20 can be found in cases of HS and MCT in Bernese Mountain Dogs and Golden Retrievers [185]. Additionally, it was shown that the presence of six top risk variants on CFA5, 11, and 14 increased the risk of the development of histiocytic sarcoma significantly [186]. Further investigations led to the discovery of some candidate genes, but no causal variants were found. Therefore, only a risk estimation using top-risk SNPs seems to be possible.

The ‘Histiocytic Sarcoma Test’ (offered by Antagene) can be performed on blood (or saliva in the case of very young puppies), and the extracted DNA is analysed to assess nine specific biomarkers (‘panel SH0912’). These biomarkers have been found to vary significantly between dogs who develop HS and those who stay healthy into old age and, as such, can be used to indicate the likelihood that the dog will develop HS. The results are presented as an index score:

- Index A: The individual has 4× the chance of not developing HS
- Index B: Neutral index
- Index C: The individual has 4× the risk of developing HS

(and an increased risk of disease markers being transferred to offspring.)

## 6. Emerging Molecular Alterations Showing Promise for Use as Biomarkers in the Clinic

With increasing genomic studies being performed on canine and feline tumour samples and germline samples, there is an increasing identification of molecular alterations that have the potential to become molecular biomarkers that are routinely used in the clinic. We review a selection of these molecular alterations that are showing potential and may soon be translated from the bench to the clinic.

### 6.1. Potential Somatic Molecular Biomarkers in Dogs and Cats

Of the various oncogenes, we chose *ERBB2* and *PIK3CA* as two likely candidates that may become important as routine tests in veterinary laboratories in the near future, as there is profound knowledge of the specific driver alterations in these genes in the cancers of dogs and cats, and targeted therapies are available for humans.

#### 6.1.1. ERBB2 (HER2) in Human Cancer

**Figure 19:**
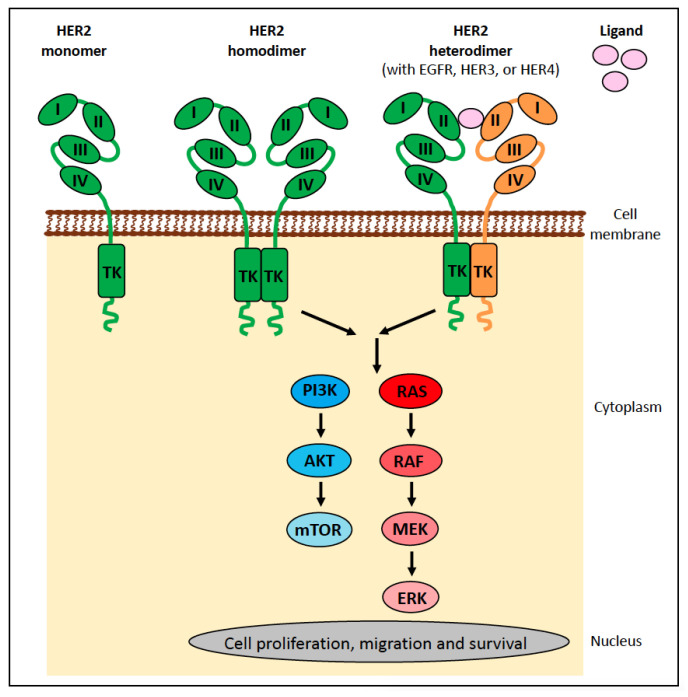
HER2 signalling. HER2 is composed of four extracellular domains (I–IV) and a cytoplasmic tyrosine kinase (TK) domain. Activation of HER2-mediated RAS/RAF/MEK/ERK and PI3K/AKT/mTOR signalling pathways occurs by heterodimerization with ligand-activated EGFR, HER3, or HER4, or by homodimerization when it is present in high concentrations. Unlike other ERBB family members, HER2 does not directly bind to any known ligands.

The *ERBB2* gene encodes HER2 (erb-b2 receptor tyrosine kinase 2, also known as human epidermal growth factor receptor 2; EGFR2). It belongs to a family of transmembrane tyrosine kinase receptors (EGFR, HER2, HER3, and HER4) that regulate several important cellular processes. These family members are activated by ligand-dependent homo-/heterodimerisation and regulate cellular proliferation and tumour progression via the downstream activation of many commonly used growth factor signalling pathways, such as the RAS/RAF/MEK/ERK and PI3K/AKT/mTOR pathways [187] (Figure 19).

**Figure 20:**
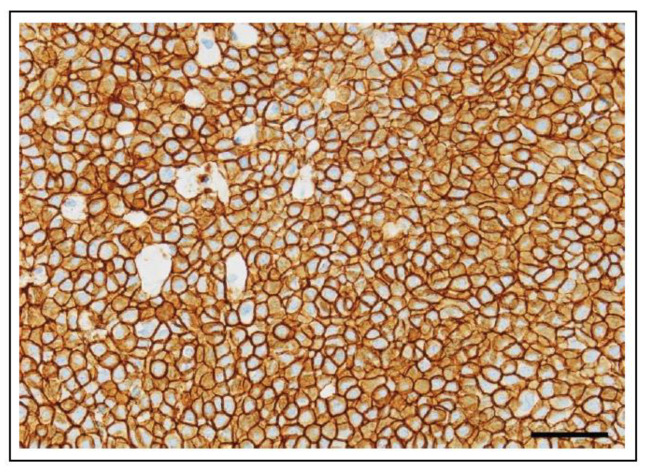
Human breast carcinoma (solid) with diffuse and strong membranous HER2 protein expression (HER2 IHC, bar = 50 µm).

*ERBB2* is overexpressed in approximately 15–20% of breast, gastric, and oesophageal human cancers as a result of amplification of the *ERBB2* gene, and this is a prognostic biomarker as “HER2 positive” tumours (which show *ERBB2* gene amplification) typically show a faster growth rate and more aggressive clinical behaviour [188]. In addition, *ERBB2* somatic mutations have been identified in *ERBB* gene-amplification-negative human breast cancers; many of these are activating mutations, and thus, *ERBB2* somatic mutation is an alternative mechanism to activate HER2 in breast cancer [189]. In human breast cancer, the level of HER2 is routinely evaluated by IHC, with in situ hybridisation used as a confirmatory test for equivocal IHC cases [190] (Figure 20).

**Figure 21:**
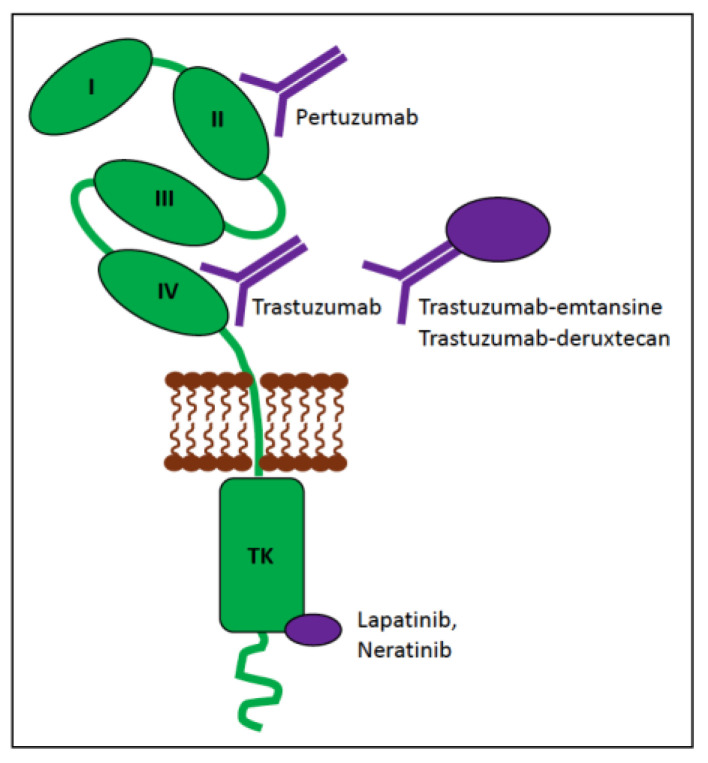
Inhibition of HER2 signalling. There are currently three types of HER2 inhibitors. (i) Trastuzumab and pertuzumab bind to HER2 at a single extracellular domain (I–IV). They exert anti-tumour effects by inhibiting downstream signalling pathways and engaging antibody-dependent cellular cytotoxicity (pertuzumab, specifically, also functions by inhibiting receptor dimerization). (ii) Trastuzumab-emtansine and trastuzumab-deruxtecan are antibody-drug conjugates targeting HER2. They exert anti-tumour effects through the same pathways mentioned above, but in addition, they show targeted cytotoxicity by releasing a highly cytotoxic agent in the vicinity of HER2-positive tumour cells. (iii) Lapatinib and neratinib are small-molecule inhibitors. They exert anti-tumour effects by binding to the intracellular tyrosine kinase (TK) domain of HER2 and directly inhibiting PI3K pathway activation.

Recently, it was realised that patients with a lower expression of HER2 might benefit from targeted therapy—not only those with an intense expression [191,192]. In human medicine, *ERBB2* gene amplification is also a predictive molecular biomarker, being the clinical criteria for the use of FDA-approved HER2-targeting drugs (such as trastuzumab, pertuzumab, neratinib, and lapatinib; Figure 21). In addition, antibody-drug conjugates (ADCs) targeting HER2 have been developed (such as trastuzumab-emtansine and trastuzumab-deruxtecan; Figure 21) and have improved OS in the second and third-line settings, with manageable adverse events [193]. As such, these drugs may offer hope for other tumour types that show *ERBB2* gene amplification, such as 2–5% of non-small cell lung cancer patients [194].

#### 6.1.2. ERBB2 (HER2) in Canine Cancer

In canines, IHC has been used to identify HER2 protein overexpression (i.e., being classified as HER2 positive) in several tumour types, including mammary carcinomas (28–40%), intestinal carcinomas (80%), rectal carcinomas (42%), and anal sac gland carcinomas (80%) [195,196,197,198]. However, only a few studies have assessed the amplification status of the *ERBB2* gene. One study found copy number gain (CNG) of *ERBB2* in canine UC (12/36, 33% cases) and polypoid cystitis (2/8, 25% cases) [199].

In canine mammary carcinoma, CNG of *ERBB2* has been reported in 10–41% of cases but was not associated with any clinical-pathological parameters [200,201]. However, one study suggested *ERBB2* CNG had potential as a prognostic biomarker, with OS in the cases with *ERBB2* CNG being significantly shorter than that in cases without *ERBB2* CNG (*p* = 0.0276) [200]. Although the *ERBB2* status of mammary tumours in canines is not yet considered in veterinary medicine, it is essential to note that trastuzumab has been shown to significantly inhibit the proliferation of canine mammary carcinoma cell lines in vitro [195].

*ERBB2* point mutations have been reported in canine pulmonary carcinomas (38% cases), with the majority (93%) being hotspot *ERBB2* p.V659E mutations, comparable to activating mutations at the same site in human cancer [202]. Notably, canine *ERBB2* p.V659E mutant pulmonary adenocarcinoma cell lines displayed a significantly higher sensitivity to the HER2 inhibitors lapatinib and neratinib relative to *ERBB2* wild-type cell lines [202]. Just recently, a pharmacokinetic study of lapatinib was performed in dogs following a single oral administration to better aid veterinarians in deciding which dose to administer [203].

#### 6.1.3. ERBB2 (HER2) in Feline Cancer

**Figure 22:**
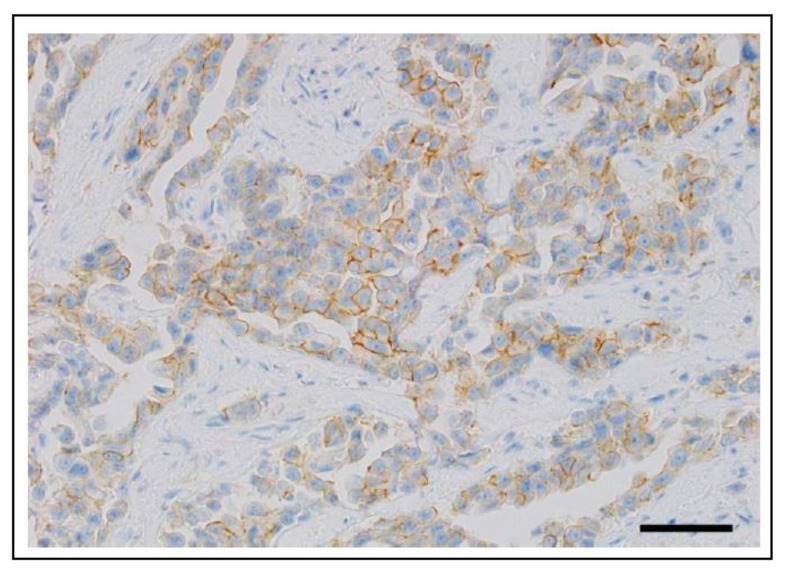
Feline mammary carcinoma demonstrating highly invasive growth. Membranous HER2 protein expression in tumour cells is present (HER2 IHC, bar = 50 µm).

In feline mammary carcinomas, IHC studies have reported over-expression of HER2 in 33–60% of cases (Figure 22) [204,205,206], which results in downstream activation of AKT [207]. HER2 overexpression correlated with a significantly shorter OS (*p* = 0.02) [204]. In contrast, *ERBB2* gene amplification in feline mammary carcinoma ranges from only 0–4% of cases [205,206].

**Figure 23:**
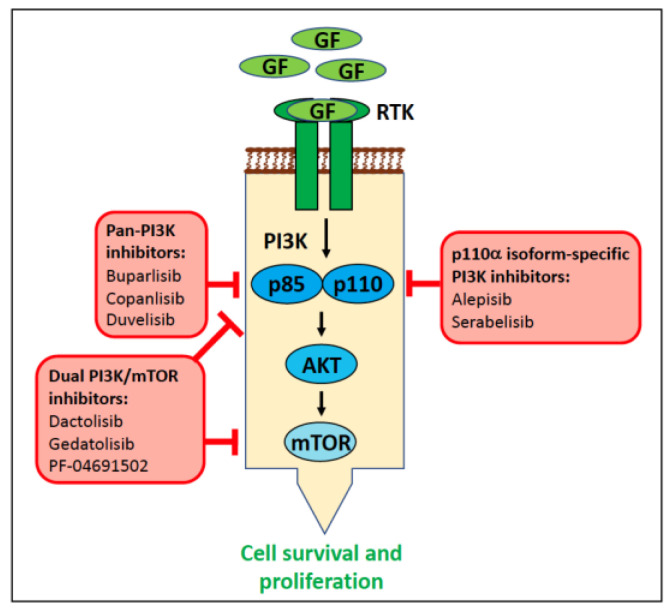
PI3K signalling and inhibition. The PI3K protein is composed of a regulatory subunit (p85) and a catalytic subunit (p110). There are three p110 isoforms: p110α (encoded by *PIK3CA*), p110β, and p110δ. PI3K signals through the AKT/mTOR downstream pathway, and this is essential for regulating multiple cellular functions. There are currently three classes of PI3K inhibitors that show activity against the p110α isoform: pan-PI3K inhibitors, dual PI3K/mTOR inhibitors, and p110α isoform-specific inhibitors.

A study of feline lung carcinomas found HER2 protein overexpression (by IHC) in 15% of cases and *ERBB2* gene amplification in 27% of cases [208]. Notably, the HER2-targeting drugs, lapatinib, neratinib, trastuzumab, and pertuzumab (Figure 23) have all been shown to induce anti-proliferative effects in feline mammary carcinoma cell lines in vitro, with synergistic effects observed when combination therapies were used [209,210]. These promising results led to a recent pharmacokinetic study of the single oral administration of lapatinib in cats, to determine plasma concentrations and thus aid veterinarians in deciding doses to use [203].

#### 6.1.4. PIK3CA in Human Cancer

Phosphatidylinositol 3-kinase (PI3K) is composed of one of three regulatory subunits (p85), all encoded by the *PIK3R1* gene, and one of three catalytic subunits, p110α, p110β, and p110δ, encoded by the *PIK3CA*, *PIK3CB*, and *PIK3CD* genes, respectively. The PI3K protein is a critical upstream regulator of the PI3K/AKT/mTOR signalling pathway (Figure 23) and is essential for regulating multiple cellular functions, including proliferation, migration, and survival. The PI3K signalling pathway is deregulated in a broad spectrum of human cancers, and *PIK3CA* is frequently mutated or amplified in many human cancers, including breast, colon, gastric, cervical, prostate, and lung cancer (reviewed in [211,212]).

Although mutations in *PIK3CA* have been found at numerous locations throughout the gene, there are hotspots at p.E542K and p.E545K (in exon 9), which function to reduce inhibition of the catalytic subunit by the regulatory subunit, and at p.H1047R (in exon 20), which serves to increase the interaction of the catalytic subunit with lipid membranes. These ‘canonical mutations’ result in PI3K hyperactivation and promote tumourigenesis via the PI3K/COX-2/PGE2 and PI3K/AKT/mTOR signalling pathways [212]. In addition to canonical and non-canonical mutations, amplification of the *PIK3CA* gene has been found in various tumour types [212]. This results in overexpression of p110α and thus increased signalling through the PI3K pathway.

Currently, three classes of PI3K inhibitors show activity against the p110α isoform (Figure 23). Specifically, pan-PI3K inhibitors (buparlisib, copanlisib, and duvelisib), dual PI3K/mTOR inhibitors (dactolisib, gedatolisib, and PF-04691502), and isoform-specific inhibitors (alpelisib and serabelisib). Each of these drugs has been tested in clinical trials. However, the results have been mixed, and toxicities are a significant concern [212]. Additional clinical trials are currently underway to further evaluate the safety and efficacy of PI3K inhibitors [212] to optimise treatment regimes, provide data on toxicities, and identify/validate predictive molecular biomarkers.

#### 6.1.5. PIK3CA in Canine Cancer

PI3K/AKT/mTOR pathway dysregulation is also very important in canine tumours. Retrospective analyses of eight different studies that performed WES on canine tumours and matched normal tissues (*n* = 1316 tumours in total) found that *TP53* and *PIK3CA* were the two most recurrently mutated genes [213]. Several studies have reported recurrent activating somatic mutations in *PIK3CA* (and *PIK3R1* to a lesser extent) in canine visceral hemangiosarcoma (HSA) [155,214,215,216,217]. *PIK3CA* is the most frequently mutated gene in canine mammary carcinomas, and mutations homologous to the *PIK3CA* hotspot mutations found in breast cancer in humans have been identified, specifically *PIK3CA* p.H1047R and p.E545K [218,219,220]. *PIK3CA* mutations have also been reported in canine oral melanoma [98], glioma [221], osteosarcoma [222,223], and canine cutaneous HSA [155].

The isoform-specific PIK3CA inhibitor, alpelisib, has been used in in vitro studies to assess the anti-tumour effects on various canine tumour cell lines and is showing promising results. One study found that alpelisib exerted a significant anti-tumour effect in *PIK3CA* mutant canine HSA cell lines but not in *PIK3CA* mutant canine mammary tumour cell lines [224]. However, another study found alpelisib exerted stronger anti-tumour effects on canine mammary tumour cell lines with *PIK3CA* mutations than on the wild-type cell lines [225]. *PIK3CA*-mutated mammary tumour organoid lines have also been shown to be more sensitive to alpelisib than wild-type organoid lines [226]. An in vitro study of the dual PI3K/mTOR inhibitors, gedatolisib and PF-04691502, tested on 12 canine tumour cell lines (of 6 tumour types), found that gedatolisib decreased cell viability in a dose-dependent manner in all cell lines; however, some were more sensitive than others [227]. However, it is interesting to note that while the presence of *PIK3CA* p.E545K and p. H1047R mutations has been shown to increase the sensitivity of tumour cells to dual PI3K/mTOR inhibitors [228], these hotspot mutations were not present in any of the 12 cell lines, indicating that they were not responsible for the increased sensitivity observed in some of the cell lines [227].

#### 6.1.6. PI3KCA in Feline Cancer

Very few molecular studies have assessed the mutational status of *PIK3CA* in feline tumours. TGS of feline cutaneous has found a *PIK3CA* mutation in 1/13 samples [155]. As more feline oncogenomic studies are performed, additional tumour types will undoubtedly have *PIK3CA* alterations (mutations or amplifications).

### 6.2. Potential Germline Molecular Biomarkers in Dogs and Cats

We chose to highlight specific tumours in which certain breeds show an increased risk of development, with a strong correlation between a molecular alteration and cancer development. These molecular alterations represent potential diagnostic biomarkers for use in the clinic.

#### 6.2.1. Potential Molecular Diagnostic Biomarkers in Dogs

Findings of strong correlations between a germline molecular alteration and increased cancer development in specific breeds are detailed in Table 1.

#### 6.2.2. Potential Molecular Diagnostic Biomarkers in Cats

Feline injection-site sarcoma (FISS) was initially documented in domestic cats in 1991 following a sudden surge in fibrosarcoma diagnoses at vaccination sites, in contrast to other locations. A significant rise in the incidence of tumours at vaccination sites was identified to have occurred between 1988 and 1994, with subsequent epidemiological investigations showing a causal link between vaccine administration and the development of tumours at injection sites [242]. A genetic predisposition was postulated because of the short latency between vaccination and tumour development and the presence of related cats. A case-control study in the USA involving 50 domestic short-hair cats with confirmed histopathologic diagnoses of FISS, matched to disease-free control cats, detected sequence variants (single nucleotide polymorphisms and insertions) in eight investigated polymorphic sites of *TP53*. The most significant association was identified for a single-base insertion (of a thymidine) in intron 7 of the gene, yielding an odds ratio of 8.99 [243]. Though the variants do not alter the amino acid sequence of TP53, the robust association between variants and disease implies a correlation. In humans, germline intronic alterations in *TP53* have been reported in patients with the rare cancer predisposition syndrome, Li-Fraumeni Syndrome, which is associated with a significantly increased risk of sarcoma and breast cancer in particular [7]. However, the correlation between the thymidine insertion and predisposition to FISS could not be confirmed in a cat population from Germany (*n* = 50) [244], suggesting further investigations are necessary.

Two studies have been carried out on mammary cancers in cats. The first study evaluated the presence of *TWIST1* gene alterations in cats with mammary cancer since a germline mutation in this gene may predispose to breast cancer in humans. Only two intronic variants were detectable. However, there was no association between the TWIST mRNA levels, the occurrence of the tumours, or the presence of these variants [245]. The second study evaluated the presence of *BRCA1* and *BRCA2* gene alterations in cats with mammary cancer as the feline tumours resemble basal-like triple-negative breast cancer in humans, which is associated with mutations in these genes. By sequencing 27 exonic regions of *BRCA1* and *2*, four germline variants in exon 9 of *BRCA1* were observed in 3/9 affected cats, and it was proposed that the germline genetic variants present in one-third of mammary carcinoma-bearing cats may be linked to an elevated risk of hereditary mammary carcinogenesis [246]. Interestingly, variants in exon 9 of *BRCA1* are significantly associated with the development of mammary tumours in dogs [234] (see Table 1).

More recently, a study performing TGS of feline cutaneous HSA and matched normal samples (*n* = 13) evaluated the presence of potential germline predisposition genes and found missense mutations in *ERCC2*, *RB1*, *IDH1*, *IDH2*, *POT1*, *TP53*, and *XPC*, with an in-frame deletion in *RB1* identified in three cases [155]. Further analysis will be needed to determine the role of these genes and molecular alterations in disease development.

## 7. Barriers to Clinical Translation of Biomarkers

In both human and veterinary medicine, there are many barriers to the successful clinical translation of biomarkers from the bench to the bedside, particularly for predictive and prognostic biomarkers (reviewed in [247]). Some of the critical considerations are discussed below, highlighting specific hurdles that are faced by veterinary medicine in contrast to human medicine. As a starting point, Table 2 provides an overview of the similarities and differences in key aspects of precision medicine between human and veterinary oncology.

### 7.1. Study Design

Inadequate sample size, resulting in a lack of statistical power, is a confounding factor in interpreting many molecular alteration study results. Indeed, underpowered studies are one of the major barriers to successful molecular biomarker validation and translation into the clinic [247]. Variables introduced during sample collection, processing, and storage are also potential sources of bias in molecular biomarker research. For example, poor-quality samples can produce false negative results, making the marker artificially appear insensitive or non-specific [247]. A lack of standardised and robust assays creates issues in interpreting the results and performing meta-analyses [247]. In contrast to conventional haematology and chemistry diagnostic biomarker tests in veterinary medicine, molecular diagnostic tests do not have a long history of benchmarking and standardisation. Thus, it is essential to develop harmonized, evidence-based best practices [248] and co-operation between different clinical laboratories/centres to allow standardisation of these processes; this will allow for increased reproducibility of study findings and downstream clinical application. For example, the European Society of Medical Oncology Precision Medicine Working Group has released recommendations for various molecular alteration-based studies, such as the use of circulating tumour DNA assays for patients with cancer [249] and germline-focussed analysis of tumour-only sequencing [250].

### 7.2. Clinical Trials

In human medicine, clinical trials are legally required for drug development and approval, however, there are several practical limitations to conducting clinical trials in veterinary medicine.

#### 7.2.1. Good Clinical Practice

‘Good Clinical Practice’ (GCP) is a set of internationally recognised scientific quality and ethical requirements that must be followed for clinical trials that involve humans. These requirements cover standards concerning all aspects of clinical trials, including their design, conduct, performance, monitoring, auditing, recording, analysis, and reporting. It provides two levels of public assurance from the study: that the results obtained are accurate and credible, and that the rights and safety of the participants are protected. In 1990, the first meeting of the ‘International Council on Harmonization (ICH) of Technical Requirements for Pharmaceuticals for Human Use’ occurred and produced guidance on GCP (https://www.ich.org/ (accessed on 23 December 2023)). In 1996, a trilateral (EU-Japan-USA) programme aimed at harmonising technical requirements for veterinary product registration was established, termed ‘International Cooperation on Harmonisation of Technical Requirements for Registration of Veterinary Medicinal Products’ (VICH). One of its five aims is to “establish and implement harmonized technical requirements for the registration of veterinary medicinal products in VICH regions” (https://vichsec.org/en/about/what-is-vich.html (accessed on 23 December 2023)). VICH continues to expand toward global support and participation, and both the ICH and VICH have been important sources of internationally accepted standards, that are often referenced in regulatory guidance documents.

#### 7.2.2. Legal Considerations

In human medicine, while the ICH’s GCP guidelines are only technically considered recommendations and thus not binding law, several elements of the guidelines have been implemented into European and US regulations relating to GCP for clinical trials [251]. In stark contrast, almost no binding regulations exist relating to conducting clinical trials in veterinary medicine. As such, the regulations used for animal experiments are typically applied, and thus the use of the animals has to be reviewed and approved by an ethical review board and associated regulatory authorities [251]. However, it needs to be appreciated that the criteria the application must meet are less strictly defined than in human clinical trials. For example, as the applications have to be conducted in accordance with regulations regarding laboratory animals, some key points are not mentioned (such as quality standards for the information provided to the owner on the consent form) and some points are not relevant (such as housing/food/environmental conditions, etc., which cannot be standardised for pet dogs as they can be for laboratory animals).

#### 7.2.3. Ethical Considerations

Transparent and honest communication between the trial co-ordinators and the participants is a key ethical aspect of clinical trials. Ideally, the participant should be provided with a detailed information sheet explaining the relevant background information, the reasons for conducting this study, any procedures that may be carried out, and any potential risks/harms [252]. In veterinary clinical trials, obtaining informed consent from the animal’s owner is a mandatory ethical requirement; however, it is substantially different from the consent obtained from an adult human participant who voluntarily decides to participate in a clinical trial. For example, there is no set standard for how much information the owner receives in veterinary clinical trials [253]. Thus, it can range from a general statement of potential implications for the animal (as required by institutional guidelines) to a detailed description of potential side effects and risks. Therefore, it can be difficult for an owner to decide whether to enroll their pet in a clinical trial. In addition, as the owner decides upon the pets treatment considering their financial situation, standardisation of the workup prior to enrolling in a trial is much more difficult than in human clinical trials.

#### 7.2.4. Sponsorship

Interdisciplinary studies between clinicians and (molecular) pathologists are necessary to evaluate the clinical benefit of the new molecular biomarkers. However, a common issue for biomarker validation studies is trial recruitment, especially if the cancer type has a low incidence/frequency. The way to address this is through large-scale, multi-centre studies. However, such trials require a huge amount of financial investment from sponsors. Despite a few rare exceptions, most trials are sponsored by pharmaceutical companies. In both human and veterinary clinical trials, companies base their decision on whether to sponsor a clinical trial first and foremost on economic considerations (profits). Obtaining backing for veterinary clinical trials is a larger hurdle than for human clinical trials for several reasons. Firstly, the number of patients who would use the treatment is far lower than in human medicine, and thus, the authorisation procedure for the use of drugs in pets is of potentially less interest to the industry. Secondly, in the veterinary setting, there is no healthcare structure with compulsory insurance, as is common for humans, so owners have to pay for the expensive clinical work up and therapeutic costs out of their own pocket. Thus, recruiting large, well-defined, randomised patient groups for clinical trials is problematic in veterinary medicine.

### 7.3. Tumour Heterogeneity

Tumour heterogeneity includes ‘intertumoural heterogeneity’ (tumour to tumour) and ‘intratumoural heterogeneity’ (ITH; within a tumour). Intertumoural heterogeneity is when primary tumours from different patients have an altered genotype and phenotype, due to diverse etiological and environmental factors. ITH is the co-existence of subpopulations of tumour cells (clones) within a given primary tumour that differ in their genetic, phenotypic, or behavioural characteristics. This is because tumour evolution is a complex biological process in which clonal lineages diverge to form distinct subpopulations. ITH can result in ‘metastatic heterogeneity’, where there is a difference in the molecular alterations between the primary tumour and circulating tumour cells (CTCs) or metastases from the same patient. For example, a study assessing the correlation of *HER2* expression between primary tumours and corresponding circulating tumour cells in human advanced breast cancer patients found non-concordant results in 32% of cases (i.e., patients with HER2-negative primary tumours had HER2-positive CTCs and vice versa) [254]. In some cases, ITH can even exceed intertumoural heterogeneity. ITH arises through a variety of mechanisms, including genetic factors, epigenetic factors, and the tumour microenvironment (including stromal and immune cells).

**Figure 24:**
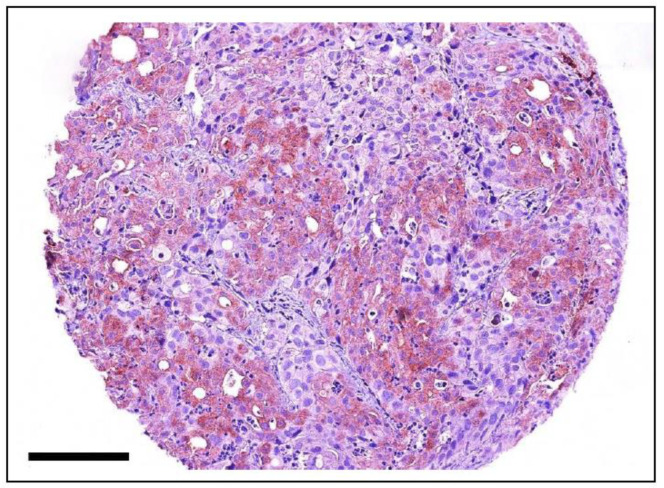
Tissue microarray core of a canine urothelial carcinoma with heterogenous mutant BRAF expression. Brown cytoplasmic labelling of tumour cells indicates the presence of mutant (V595E) BRAF protein, which is considered absent in tumour cells with negative staining (BRAFV600E immunohistochemistry, bar = 100 µm).

Although both types of heterogeneity can make the identification of molecular biomarkers rather challenging, ITH poses a ‘technical’ issue. This is because many tests involve analysis of the tumour ‘bulk’, such as a biopsy sample of the tumour which contains numerous tumour cells, as opposed to analysis of single tumour cells. Thus, they cannot assess the proportion of the tumour cells that are ‘positive’ for the molecular biomarker. For example, a study which performed mutant BRAF IHC on canine urinary bladder UC showed that *BRAF* p.V595E mutant tumours (as determined by ddPCR) did not show mutant BRAF immunopositivity across the whole section of the tumour [47] (Figure 24).

This poses a problem as the ‘representativity’ of a sample varies depending on the sampling method used (e.g., liquid biopsy, fine needle aspiration (cytology), true cut biopsy, tumour mass resection, etc). Tests based on a single small sample may only capture a ‘snapshot’ of the genomic diversity present within the entirety of the tumour [247]. Minimally invasive techniques such as liquid biopsies can allow serial sampling. However, ITH and metastatic heterogeneity can affect treatment response, as subpopulations can vary widely in their response to therapeutic agents, and incomplete treatment responses (due to clonal diversity) can result in the acquisition of resistance [255]. Thus, tumour heterogeneity poses a tremendous challenge for precision medicine and the identification of molecular biomarkers and their translation into the clinic.

### 7.4. Complexity of Cellular Oncogenic Mechanisms

A reality of oncology is that, despite being positive for a particular predictive biomarker and the appropriate therapy chosen, the patient may only show a poor response occasionally. This ‘resistance’ to the therapeutic agent (as described above for TKIs) could be due to incomplete/partial pathway inhibition by the therapeutic agent, activation of compensatory pathways, the presence of co-occurring driver mutations in the tumour, and/or ITH (a small proportion of tumour cells lacking the targeted biomarker can outgrow the biomarker-positive tumour cells that are being killed by the targeted therapy). Thus, the assessment of a single biomarker may not be sufficient. Co-occurring mutations and/or signalling pathways may also need to be investigated to allow for combination-targeted therapy regimes. Thus, it is recommended that molecular characterisation of the newly developed metastases be performed (such as by less invasive methods, e.g., by liquid biopsy or cytology) or that the molecular characterisation of the recurrent nodule be investigated after surgical removal. However, in veterinary medicine, the owners often decide against further diagnostics and therapy in cases of metastases or relapse, typically for financial and/or ethical reasons.

## 8. Conclusions

Despite the hurdles that must be overcome with molecular biomarkers to firstly identify a relevant molecular alteration and secondly translate it into clinical use, we have shown that there are, at present, a range of molecular alterations that have become widely used clinical molecular biomarkers. Indeed, it is encouraging to see the diagnostic, prognostic, and predictive benefits that these assays to detect specific molecular alterations are affording our pet dogs and cats with cancer.

Of course, as with genetic testing in human medicine, there are some limitations to these molecular alteration assays. For example, some samples may be too small to be able to obtain sufficient DNA for analysis and/or generate accurate results (such as ctDNA in liquid biopsy samples if the tumour does not shed into the bloodstream). In addition, tests that interrogate one gene may miss identifying other genes (biomarkers), impacting diagnostic, prognostic, and/or predictive outcomes. The same applies to multi-gene panels if the relevant gene/genomic region is not on the panel. Finally, intra-tumoural heterogeneity, as well as the evolution of a tumour over time, can be a confounding factor, particularly when molecular biomarkers are used for prognostic or predictive purposes. Nevertheless, precision medicine has unquestionably revolutionised the field of oncology in human medicine. Thus, with the increasing availability of clinical tests for detecting somatic and germline molecular alterations in canines and felines, the future of cancer care for our pet dogs and cats has never looked brighter.

## Figures and Tables

**Table 1:** Potential molecular diagnostic biomarkers for tumour predisposition in canines (alphabetical order). Abbreviations: BMD, Bernese Mountain Dog; CFA, *Canis familiaris* chromosome; ESS, English Springer Spaniel; FCR, Flat-coated Retriever; GR, Golden Retriever; HSA, hemangiosarcoma; HS, histiocytic sarcoma; MCT, mast cell tumour; OSA, osteosarcoma.

Tumour Type	Affected Breed(s)	Molecular Alteration	Ref
B-cell lymphoma	BMD, GR, FCR	Two loci on CFA5	[186,229,230]
Gastro-intestinal polyposis	Jack Russell Terrier (in Japan)	2-bp substitution in *APC.* The second base pair substitution is a missense mutation (*APC* p.K155X), resulting in a truncated protein. Transgenerational transmission of hereditary gastrointestinal polyposis associated with the germline *APC* variant has been observed in Jack Russell Terrier lineages in Japan	[231,232]
HS	GR, BMD	Haplotypes harbouring the hyaluronidase genes *HYAL1*, *HYAL2*, and *HYAL3* on CFA20	[186]
HSA	GR	Two loci on CFA5 (same loci as B-cell lymphoma)	[229]
Mammary tumour	ESS and a variety of breeds	SNPs in *BRCA1* and *BRCA2*	[233,234,235,236]
Mammary tumour	ESS	Haplotype on CFA11 spanning a 446 kb region, overlapping *CDK5RAP2* and two loci on CFA27	[237]
MCT	GR	SNP in *GNAI2*, introducing an alternative splice form of the gene and resulting in a truncated protein in European and US populations. Haplotypes harbouring the hyaluronidase genes *HYAL1*, *HYAL2*, and *HYAL3* on CFA20 in European populations. Haplotypes harbouring the hyaluronidase genes *HYAL4*, *SPAM1*, and *HYALP1* on CFA14 in US populations	[185]
MCT	BMD	Haplotypes harbouring the hyaluronidase genes *HYAL1, HYAL2* and *HYAL3*, on CFA20	[186]
MCT (skin)	GR, Labrador Retriever	Synonymous variant in *DSCAM* on CFA31	[238]
OSA	Scottish Deerhound	Haplotype on CFA34	[239]
OSA	Greyhound, Leonberger	A rearranged locus 150 kilobases upstream of *CDKN2A*/B on CFA11 alters the regulation of downstream genes	[240,241]

**Table 2:** Overview of key aspects of precision medicine and the differences currently seen in human and veterinary oncology.

Comparator	Human Medicine	Veterinary Medicine	Veterinary Medicine Hurdles
Techniques used in routine diagnostics	A broad spectrum of techniques are available.	Mainly PCR/ddPCR. Sequencing is starting to be used, but there has been no analysis of methylation or translocation status.	The stage of research is about 20 years behind that of human medicine.
Use of liquid biopsies	There is a lot of research on ctDNA monitoring of patients with specific tumour types and liquid biopsies routinely used in the clinic.	Very few ctDNA monitoring studies in dogs and none in cats. Liquid biopsies are not routinely used in the clinic.	Limited knowledge about genetic alterations of the tumours exists (the use of liquid biopsies is in an early stage of research).
Use of multi-gene sequencing panels in routine diagnostics	Multiple panels are available and routinely used in the clinic.	A few panels are currently available, but they are not part of routine diagnostics.	Financial limitations because owners have to pay and the tests are expensive.
Use of somatic alterations found in tumours as molecular biomarkers in the clinic	Numerous diagnostic, prognostic, predictive, and screening molecular biomarkers are clinically available for specific tumour types (eg., lung, breast, colorectal, and thyroid cancer).	Only two are available in the clinic: *BRAF* (used as a diagnostic and predictive biomarker, also used in a monitoring setting) and *KIT* (used as a prognostic and predictive biomarker).	Limited knowledge about genetic alterations of the tumours, and limitations in study design and availability of clinical trials to allow translation of research findings to the clinic. The cost to the owner is also an issue.
Use of germline alterations as molecular biomarkers in the clinic	Numerous molecular biomarkers are routinely used in the clinic for screening and monitoring patients (e.g., *BRCA1/2*).	Some germline variants have been identified as risk factors and may be used when considering breeding aspects.	Pedigrees must be available. The cost to the owner is also an issue.
Treatment opportunities available	Tumour boards, standardized therapeutic concepts, and intense monitoring of the patients with first, second, and third-line therapies in case of recurrence or resistance in specific tumour diseases.	Rarely do tumour boards exist, lack of uniform protocols for the management of cancer, and limited knowledge on dosage/effectivity/side effects of new drugs (or when using human drugs off-label).	Very high costs for the owners, limited availability of new drugs (even limited availability of access to chemotherapy and radiation therapy).
State of clinical studies/research	Existence of cancer centres and cancer registries. Numerous medical universities are carrying out clinical research. Clinical studies conducted by the pharmaceutical industry.	There are no cancer centers or cancer registries. There are few veterinary universities and no sponsorship of clinical studies by the pharmaceutical industry.	There are few studies involving canine tumours, even less feline tumours. Poor compliance by the owners. Follow-up data are very difficult to collect.
Financial aspects	Sponsoring of research/clinical trials by pharmaceutical industry. Government subsidisation (and/or private insurance coverage) of some diagnostic and treatment costs after the establishment of the method.	There is a very small investment by pharmaceutical industry into sponsoring research/clinical trials. There is no government subsidisation of any costs, and pet insurance only offers limited coverage of diagnostic/treatment costs (and often does not cover precision medicine-related costs as not yet considered ‘routine’).	The owner may not be able to afford the expensive diagnostic tests/treatments and thus decide to euthanise the pet instead of using precision medicine.
Ethics	Rules exist regarding patient counselling, patient data confidentiality, ensuring the patient’s best interests, etc. The patient decides on the use of precision medicine after discussion with the clinician.	No defined rules on patient conselling and data confidentiality. The owner decides on the use of precision medicine based on financial and personal reasons, which may not necessarily be in the animal’s best interests.	Technical and financial limitations. There are also limitations in terms of the education of veterinarians about the field of precision medicine (due to its new/emerging/nature).

## Data Availability

All data contained within the article.

## Abbreviations

ADC	antibody-drug conjugate
AI	artificial intelligence
AKT	protein kinase B
BMD	Bernese Mountain dog
BRAFi	BRAF inhibitors
cfDNA	cell-free DNA
ctDNA	circulating tumour DNA
CFA	*Canis familiaris* chromosome
CNA	copy number alteration
CNG	copy number gain
CNV	copy number variation
ddPCR	droplet digital PCR
DSCC	squamous cell carcinoma of the digit
EMA	European Medicines Agency
ERBB2	erb-b2 receptor tyrosine kinase 2
ERK	extracellular signal-regulated kinase
ESS	English Springer Spaniel
FCR	Flat-coated Retriever
FDA	United States Food and Drug Administration
FFPE	formalin-fixed paraffin-embedded
FFTC	familial follicular thyroid carcinoma
FISS	Feline injection-site sarcoma
FTC	follicular thyroid carcinoma
GCP	Good Clinical Practice
GEP	gene expression profile
GOF	gain of function
GIST	gastrointestinal tumour
GR	Golden Retriever
GS	Giant Schnauzer
GSD	German Shepherd dog
GWAS	genome-wide association study
HE	hematoxylin and eosin
HS	histiocytic sarcoma
HSA	hemangiosarcoma
ICH	International Council on Harmonization
IgH	immunoglobulin heavy chain
IHC	immunohistochemistry
ITD	internal tandem duplications
ITH	intratumoural heterogeneity
JAK	Janus kinase
JM	juxtamembrane
MAPK	mitogen-activated protein kinase
MCED	multi-cancer early detection
MCT	mast cell tumour
MEK	mitogen-activated protein kinase kinase
MNV	multi-nucleotide variant
MSKCC	Memorial Sloan Kettering Cancer Center
NGS	next-generation sequencing
OS	overall survival
OSA	osteosarcoma
PARR	PCR for antigen receptor rearrangement
PC	prostate carcinoma
PCR	polymerase chain reaction
PI3K	Phosphatidylinositol 3-kinase
PFS	progression-free survival
PLCγ	phospholipase C-γ
PODS	Precision Oncology Decision Support
RAF	rapidly accelerated fibrosarcoma
RCND	renal cystadenocarcinoma and nodular dermatofibrosis
RTK	receptor tyrosine kinase
SCC	squamous cell carcinoma
SCF	stem cell factor
SNV	single nucleotide variation
SS	Standard Schnauzer
STAT	signal transducer and activator of transcription
STPO	standard poodle
TCR	T-cell receptor
TGS	targeted sequencing
TSG	tumour suppressor gene
TK	tyrosine kinase
TKI	tyrosine kinase inhibitor
UC	urothelial carcinoma
VICH	International Cooperation on Harmonisation of Technical Requirements for Registration of Veterinary Medicinal Products’
VUS	variants of unknown significance
WES	whole exome sequencing
WGS	whole genome sequencing
